# All-solution-processed scalable and wearable organic thermoelectrics by structurally mimicking transverse thermoelectric effects

**DOI:** 10.1126/sciadv.aea9094

**Published:** 2026-03-18

**Authors:** Juhyung Park, Sun Hong Kim, Jeong Han Song, Jeehyun Jeong, Jeonghun Kwak

**Affiliations:** ^1^ Department of Electrical and Computer Engineering, Seoul National University, 1-Gwanak-ro, Gwanak-gu, Seoul 08826, Republic of Korea.; ^2^ Department of Chemical Engineering, University of Seoul, 163 Seoulsiripdaero, Dongdaemun-gu, Seoul 02504, Republic of Korea.; ^3^ Inter-university Semiconductor Research Center and SOFT Foundry Institute, Seoul National University, 1-Gwanak-ro, Gwanak-gu, Seoul 08826, Republic of Korea.

## Abstract

Organic thin-film thermoelectric generators (TEGs) hold great promise as flexible and wearable energy harvesters. However, their broader application is hindered by the difficulty of establishing a temperature gradient within the thin film when using planar heat sources. Here, we introduce pseudo-transverse TEGs (pT-TEGs), an architecture employing an elastic substrate with two thermal conductivities, enabling the orthogonal conversion of longitudinal heat flux into lateral thermovoltage by mimicking the transverse thermoelectric effect. This design circumvents the limitations of conventional thin-film TEGs, such as the need for three-dimensional deformation to form a temperature gradient, allowing efficient thermal energy harvesting from planar heat sources while preserving a two-dimensional form factor. Furthermore, the fully solution-processed and modular pT-TEGs exhibit excellent mechanical flexibility and high scalability. We believe that this approach offers a practical platform for the realization of genuine two-dimensional TEGs and will contribute to the advancement of flexible and wearable energy harvesting technologies.

## Teaser

A flexible organic device harvests body heat by converting vertical thermal gradients into useful lateral electrical energy.

## INTRODUCTION

The rapid advancement of soft wearable electronics (*1*, *2*) has accelerated the demand for self-powered technologies that continuously harvest energy from the ambient environment (*3*–*6*). Thermoelectric (TE) energy harvesting offers an effective solution by supplying a direct current to devices by exploiting the constant thermal gradient generated by the human body (*7*), even without mechanical motion (*8*, *9*). Considerable efforts have been devoted to developing flexible and wearable thermoelectric generators (TEGs) for this purpose (*10*–*14*). In particular, thin-film TEGs using organic semiconductors show substantial promise as wearable power sources (*15*–*19*), offering advantages such as reduced weight (*20*), lower toxicity (*21*, *22*), cost-effectiveness (*23*), and facile fabrication when compared with those of TEGs based on bulkier inorganic compounds (*24*–*26*). In addition, their intrinsically thin and flexible nature (*27*) allows excellent conformity to curved surfaces, such as human skin (*28*), thereby minimizing the thermal resistance between the heat source and the device (*29*).

Despite these advantages, thin-film TEGs face a major hurdle: establishing a robust longitudinal temperature difference (∆*T*) when placed on a planar heat source (*30*–*32*). This issue arises primarily due to the extremely small TE layer thickness (typically below a few micrometers), which restricts the effective utilization of the Seebeck effect along the heat flow direction, from the heat source surface to the ambient air (*33*). To date, several methods have been introduced to address this issue, including vertical arrangement of flat devices (*34*), transformation of the two-dimensional (2D) structure into a 3D one (*35*), using kirigami or origami techniques (*36*, *37*), or employing 3D printing (*8*), all of which result in a longitudinal ∆*T* (∆*T*_⊥_). Ironically, these strategies not only entail structural distortion of the TE elements but also increase the volume of the thin-film device, thereby undermining its inherent 2D advantages. To realize a true 2D thin-film TEG, it is essential to produce an in-plane ∆*T* (∆*T*_∥_) and resulting thermovoltage (∆*V*) on a planar heat source with ∆*T*_⊥_. From this perspective, we first directed our attention to transverse TE effects, which convert ∆*T*_⊥_ into in-plane electrical current (*38*, *39*). The Nernst effect (*40*), which leverages the magnetization of certain materials [e.g., ferromagnetic metals such as iron (*41*) and magnetic Heusler alloys such as Co_2_MnGa] (*42*), and the spin Seebeck effect, which occurs in a bilayer comprising a magnetic material and a normal metal with strong spin-orbit coupling (*43*, *44*), are representative examples of transverse TE effects. Owing to the orthogonal relationship between ∆*T* and electric flux (*45*–*47*), these approaches offer the potential to overcome the aforementioned limitations, allowing for a simplified device structure with straightforward wiring (*48*). However, these phenomena have been demonstrated exclusively not only in certain types of inorganic materials (*40*) and their hybrid composites (*49*) but also the transverse thermopower (typically <10 μV K^−1^) lags behind the voltage levels obtainable from the ordinary Seebeck effect (*38*). Consequently, there is an urgent need to develop an innovative strategy capable of generating an in-plane ∆*V* orthogonal to the direction of heat flow to fully leverage the potential of thin film–based TEGs.

Here, we propose a thin-film architecture for flexible, wearable, and scalable TEGs capable of generating in-plane ∆*V* from a planar heat source by structurally mimicking the transverse TE effects. To realize this, an elastic substrate composed of two regions with distinct thermal conductivities (κ) is developed, enabling the conversion of ∆*T*_⊥_ into ∆*T*_∥_. This induces a temperature gradient across the thin organic TE layer, thereby generating an in-plane ∆*V* orthogonal to the original direction of heat flow. As this mechanism is theoretically distinct while structurally mimics transverse TE effects, we term it a “pseudo-transverse” TEG (pT-TEG). All components of the pT-TEG—including the elastic dual-κ substrate, organic TE layer, and elastomeric electrodes—are solution-processed and intrinsically flexible. In addition, through the chessboard-patterned modular design strategy, we demonstrate an assembly-oriented, multiscale pT-TEG modules comprising up to eight pairs of p-n legs in various shapes. This genuine 2D configuration enables effective heat-to-electricity conversion on arbitrarily curved heat sources, offering excellent compatibility with wearable electronics.

## RESULTS

### Design of pT-TEGs with a dual-κ substrate

**Fig. 1.**
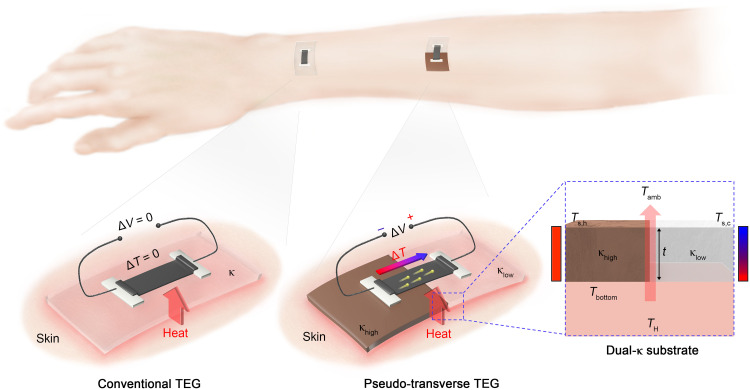
Comparison between conventional TEG and pT-TEG. A conventional thin-film TEG placed on human skin (i.e., a planar heat source) cannot produce ∆*T* and ∆*V* in a longitudinal direction, whereas the pT-TEG with the dual-κ substrate can produce in-plane ∆*T* and ∆*V* by redirecting the heat flow. The figure also illustrates the annotations for the thermal conductivity and thickness of the substrate, as well as the temperature for each location.

Figure 1 presents a schematic illustration comparing the conventional thin-film TEGs with our pT-TEGs. Conventional thin-film TEGs, when attached to a heat source such as human skin, are unable to create ∆*T* in the in-plane direction and therefore fail to generate ∆*V* between two electrodes positioned in parallel. In contrast, our pT-TEGs can spontaneously convert ∆*T*_⊥_ into ∆*T*_∥_, thereby inducing an electric potential difference in the in-plane direction, effectively mimicking transverse TE effects. This unique function in our pT-TEGs is achieved through the development of a “dual-κ substrate,” which is composed of two elastic materials with different thermal conductivities, one with higher κ (κ_high_) than the other (κ_low_), placed side by side, as illustrated in Fig. 1. In accordance with Fourier’s law (*Q* = −κd*T*/d*t*, where *Q* is the local heat flux density and *t* is the thickness of the substrate), this design results in a higher surface temperature (*T*_s_) in the κ_high_ region (*T*_s,h_) than the κ_low_ region (*T*_s,c_) by redirecting the heat flow. Through a systematic study in which κ_high_, κ_low_, and thickness of the dual-κ substrate were varied, we observed that *T*_s,h_ and *T*_s,c_ can readily approach the heat source temperature (*T*_H_) and the ambient temperature (*T*_amb_), respectively. This substrate-driven thermal engineering enables the formation of a maximum ∆*T*_∥_ close to *T*_H_ − *T*_amb_ within a 2D structure. Furthermore, the use of elastic materials in the dual-κ substrate facilitates conformal contact with irregular or dynamic surfaces such as human skin, effectively reducing thermal resistance at the interface between the heat source and the pT-TEGs.

**Fig. 2.**
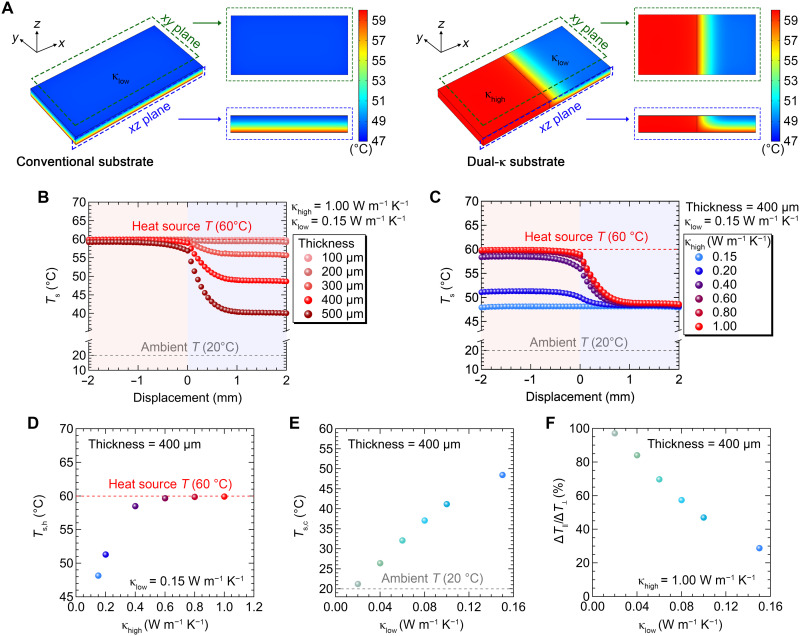
FEA simulations on heat transfer of the substrates. (**A**) Temperature distribution of the conventional and dual-κ substrates. *T*_H_ and *T*_amb_ are fixed to 60° and 20°C, respectively. The *T*_s_ profile near the junction of the dual-κ substrate with different (**B**) thickness (100 to 500 μm) and (**C**) κ_high_ (0.15 to 1.00 W m^−1^ K^−1^) when κ_low_ is fixed to 0.15 W m^−1^ K^−1^. (**D**) *T*_s,h_ as a function of κ_high_. The *T*_s,h_ value approaches to *T*_H_ when κ_high_ is 0.60 W m^−1^ K^−1^ or higher. (**E**) *T*_s,c_ as a function of κ_low_, which converges to the *T*_amb_ when κ_low_ decreases to 0.02 W m^−1^ K^−1^. (**F**) Ratios of ∆*T* in the parallel direction to that in the longitudinal direction as a function of κ_low_.

First, to optimize the design of a dual-κ substrate for effective in-plane ∆*T* generation, we calculated the temperature distribution through heat transfer simulations using finite element analysis (FEA), varying the conditions. The detailed simulation methods are described in Materials and Methods. Initially, the values of κ_low_ and κ_high_ were set to 0.15 and 1.00 W m^−1^ K^−1^, respectively, based on our experimental data shown in the following section. The substrate thickness was fixed to 400 μm. Figure 2A presents the simulation results, revealing that the dual-κ substrate produces ∆*T*_∥_ of 11.2°C on the surface when attached to a heat source of 60°C (ambient air = 20°C for simulation), whereas the conventional substrate generates no in-plane ∆*T*. This ∆*T*_∥_ originates from the different heat transfer capabilities along the *z* axis within each region of the dual-κ substrate, as confirmed by the cross-sectional view (*xz* plane in Fig. 2A) and the depth profile shown in fig. S1A.

Following this initial validation, we systematically investigated how the geometrical and thermal properties of the dual-κ substrate influence heat transfer and *T*_s_ in each region. Figure 2B shows the calculated surface temperature near the κ_high_/κ_low_ interface for various substrate thickness. Notably, when *t* = 100 μm or less, *T*_s_ in both regions becomes nearly identical, and thus no Δ*T*_∥_ is formed. This indicates that 100 μm is the minimum required thickness for Δ*T*_∥_ formation under the given κ values. When *t* increases to 500 μm, Δ*T*_∥_ rises sharply, reaching 19.2°C, which is primarily attributed to the excellent thermal insulation in the κ_low_ region (fig. S1, A and B). We limited the substrate thickness to a maximum of 500 μm due to the inherent trade-offs between thickness and mechanical flexibility. Subsequently, we evaluated the *T*_s_ profile near the junction by varying the κ_high_ value from 0.15 to 1.00 W m^−1^ K^−1^, as shown in Fig. 2C, while the thickness is fixed to 400 μm. It is evident that Δ*T*_∥_ begins to emerge once κ_high_ exceeds κ_low_. As κ_high_ increases, *T*_s,h_ rises markedly, whereas *T*_s,c_ remains nearly unchanged, resulting in an enhanced ∆*T*_∥_, as shown in Fig. 2C and fig. S1C. When κ_high_ is 0.60 W m^−1^ K^−1^ or higher, *T*_s,h_ closely approaches the temperature of the heat source (60°C), leading to a plateau in ∆*T*_∥_ at ~11.2°C (Fig. 2D). In parallel, we examined the effect of κ_low_ on *T*_s,c_. As shown in Fig. 2E, a reduction in κ_low_ led to a corresponding decrease in *T*_s,c_, which ultimately approached the ambient temperature of 20°C when κ_low_ was lowered to 0.02 W m^−1^ K^−1^. This finding implies that, when κ_low_ and κ_high_ are set to 0.02 and 1.00 W m^−1^ K^−1^, respectively, nearly the entire ∆*T*_⊥_ can be converted into ∆*T*_∥_, as depicted in Fig. 2F. The dependence of ∆*T*_∥_ on boundary conditions, such as the heat transfer coefficient (*h*) and heat source temperature *T*_H_, is further summarized in fig. S2. Having established the optimized conditions for ∆*T*_∥_ generation via FEA simulations, we next turn our attention to the fabrication and performance characterization of pT-TEGs on the dual-κ substrate.

### Fabrication and performance characterization of pT-TEGs

To realize a single substrate with dual κ, it is crucial to use a material that is physically homogeneous yet allows for the local tuning of κ to form a distinct thermal junction. For this purpose, a silicone elastomer, polydimethylsiloxane (PDMS), was selected. Crucially, its curing process can be precisely controlled to enable seamless integration of two regions with different thermal conductivities, resulting in a monolithic substrate that visually and functionally behaves as a single continuous unit. Moreover, PDMS is compatible with solution-based fabrication and can be readily blended with other materials to form composites, allowing further adjustment of its thermal properties. It is also optically transparent, chemically inert, nontoxic, and stretchable, making it an ideal material for wearable TEGs.

**Fig. 3.**
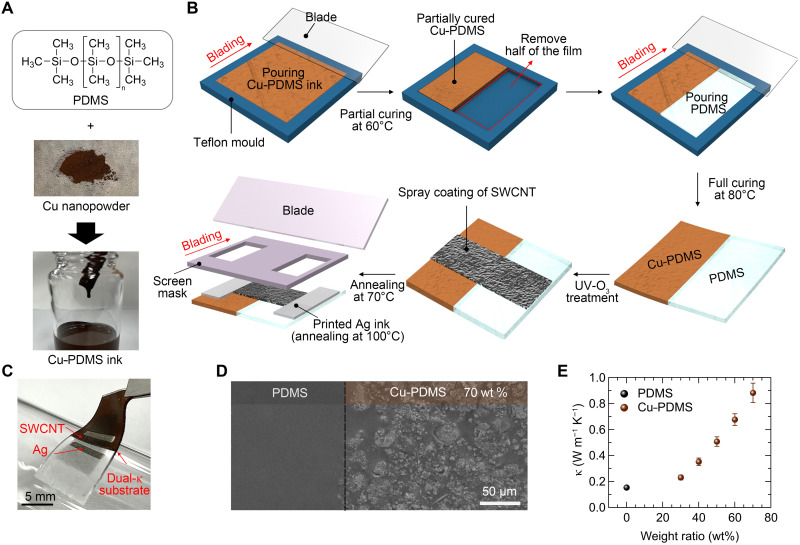
Fabrication process of the dual-κ substrate and pT-TEG. (**A**) Schematic illustration of the fabrication process of the Cu-PDMS composite ink and (**B**) the SWCNT-based pT-TEG. (**C**) Photograph of the flexible pT-TEG. (**D**) Cross-sectional SEM images of the dual-κ substrate near the junction between PDMS and Cu-PDMS (70 wt %). (**E**) Thermal conductivity of the pristine PDMS and the Cu-PDMS substrates as a function of the weight ratio of Cu nanopowder.

To form the κ_high_ region, metallic Cu nanopowder was blended into PDMS to obtain a thermally conductive Cu-PDMS composite ink (Fig. 3A). For the κ_low_ region, pristine PDMS was used due to its inherently low κ (0.15 W m^−1^ K^−1^) (*50*). Figure 3B illustrates the fabrication process of the dual-κ substrate and the pT-TEG. Specifically, the Cu-PDMS composite ink was first cast into a Teflon mold and bladed to form a 400-μm-thick film. After partially curing at 60°C, half of the film was removed and replaced with pure PDMS via the same blading method. The partial curing of the Cu-PDMS at a low temperature retained its shape during the pristine PDMS deposition and facilitated robust bonding at the interface through polymerization with full curing at 80°C. The electrical insulation of the Cu-containing area must be preserved to allow direct deposition of the TE active layer. The Cu-PDMS composite was confirmed to be thermally conductive yet electrically insulating within the miscible limit [~70 weight % (wt %)], attributed to the absence of continuous electrical percolation paths among the Cu nanoparticles, as discussed in the following section. On the prepared dual-κ substrate, single-walled carbon nanotubes (SWCNTs) were spray-coated as the p-type TE active layer. SWCNTs were chosen for their compatibility with solution-printed flexible fabrication, which enables clean patterning and scalable processing on the dual-κ platform. In addition, they exhibit high-power factors and, unlike many conjugated polymers, can be converted to n-type through mild amine treatments [e.g., polyethyleneimine (PEI)]. This allows the use of the same material for both p- and n-type legs with competitive performance and good stability, particularly under ambient conditions (*51*, *52*). Last, intrinsically stretchable Ag composite ink was screen-printed as the contact electrodes. Note that the device geometry, including the electrode spacing, was determined on the basis of the thermal-circuit model and the thermal healing-length criterion (see note S1 and fig. S3) (*53*). A photograph of the SWCNT-based flexible pT-TEG fabricated on the dual-κ substrate is presented in Fig. 3C.

The internal morphology of dual-κ substrate was investigated using cross-sectional scanning electron microscopy (SEM). To prepare homogeneous Cu-PDMS composites, Cu nanopowder with an average particle size of 800 nm was mixed into PDMS at weight ratios of up to 70%, followed by sufficient mechanical mixing. When the Cu content exceeded 70 wt %, the resulting Cu-PDMS composite films showed poor elasticity, as shown in fig. S4. Although the Cu nanopowder tended to locally aggregate into clusters ranging from several to tens of micrometers in size, the composites overall exhibited uniform dispersion without apparent phase separation, as confirmed in fig. S5. Consequently, it was possible to control the κ without losing electrical insulation properties. We also examined the junction region of the dual-κ substrate. Figure 3D shows a well-defined boundary between the pristine and the Cu-containing PDMS regions, with no swelling or delamination, confirming that the two regions remain seamlessly integrated as a single, continuous substrate. The robustness of the Cu-PDMS/PDMS interface was demonstrated through a quantitative evaluation of its interfacial fracture energy (see note S2 and fig. S6 for details) (*54*).

Next, we characterized the change in κ of the Cu-PDMS composites as a function of Cu loading. The κ values of PDMS and Cu-PDMS substrates were measured by the laser flash method based on the equation κ = *DC*_p_ρ, where *D*, *C*_p_, and ρ are the thermal diffusivity, specific heat, and density, respectively. As plotted in Fig. 3E, increasing the Cu nanopowder concentration led to a proportional enhancement in the κ of the Cu-PDMS substrate. In particular, at 60 and 70 wt % Cu loading, the thermal conductivities were measured to be 0.68 and 0.88 W m^−1^ K^−1^, respectively. These values are sufficient to raise the surface temperature of the κ_high_ region close to that of the heat source, in agreement with the FEA simulation results shown in Fig. 2D.

**Fig. 4.**
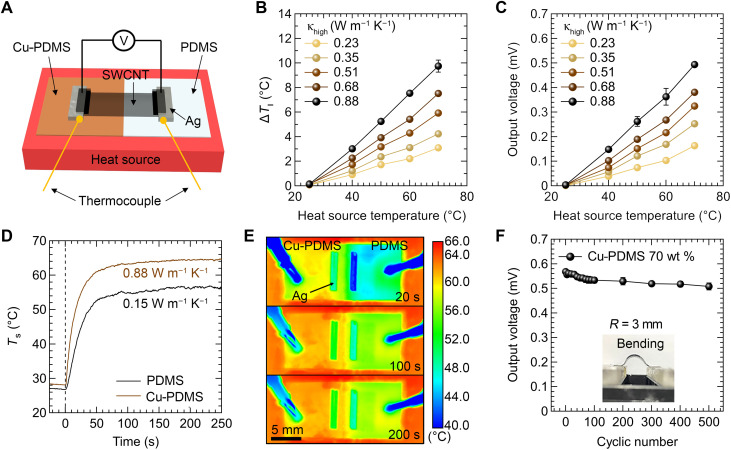
Performance of SWCNT-based pT-TEGs. (**A**) Measurement setup for evaluating the TE performance of the pT-TEG placed on a single planar heat source. (**B**) ∆*T*_∥_ and (**C**) output voltage of the pT-TEGs as a function of *T*_H_ at various κ_high_ values. (**D**) *T*_s_ of the Cu-PDMS and pristine PDMS regions over time when *T*_H_ is 66°C. The dashed line marks the onset of heating. (**E**) Surface temperature distribution captured by an IR camera at 40, 70, and 150 s after heating. (**F**) Output voltage of the pT-TEG as a function of the number of bending cycles. The bending radius was 3 mm.

To verify that the pT-TEGs effectively form ∆*T* on the surface of the dual-κ substrate, we placed the device on a single Peltier module serving as a planar heat source, as shown in Fig. 4A. This setup differs from the conventional configuration for planar TE devices, which typically uses two Peltier modules positioned side by side. Then, we measured the in-plane temperature difference between the κ_low_ and κ_high_ regions on the pT-TEG surface (∆*T*_∥_ = *T*_s,h_ − *T*_s,c_) as a function of the heat source temperature (*T*_H_) while varying κ of the Cu-PDMS composite. As shown in Fig. 4B, ∆*T*_∥_ increases almost linearly with rising *T*_H_ across all Cu concentrations. Moreover, for a fixed *T*_H_, higher κ_high_ values resulted in larger ∆*T*_∥_. According to the simulations in fig. S3B, our achieved κ_high_ of 0.88 W m^−1^ K^−1^ already lies near the regime where ∆*T*_∥_ saturates. Even when κ_high_ is artificially increased to extreme values, the additional improvement in ∆*T*_∥_ is minimal, indicating that our Cu-PDMS formulation provides a practically optimized thermal contrast for maximizing ∆*T*_∥_. The resultant ∆*T*_∥_ established within the SWCNT film induces Δ*V* via the Seebeck effect, which follows the same trend as ∆*T*_∥_, as plotted in Fig. 4C. For example, the output voltage reached a maximum value of ~494 μV at *T*_H_ of 70°C with κ_high_ of 0.88 W m^−1^ K^−1^, corresponding to ∆*T* of 9.8°C. For this composition, the Seebeck coefficient (α), calculated using the equation α = ∆*V*/∆*T*_∥_ from the data in Fig. 4 (B and C), was ~50.3 μV K^−1^ for the pT-TEG. It is worth noting that this value is in close agreement with that obtained from the p-type SWCNT-based TE device using a conventional device architecture (α = 52.5 ± 4.09 μV K^−1^), as shown in fig. S7A. Furthermore, across all Cu-PDMS composites, the extracted Seebeck coefficients remain nearly constant with an average of 53.96 ± 4.03 μV K^−1^, indicating no systematic dependence on Cu content (fig. S7B). These results demonstrate that the proposed device architecture based on the dual-κ substrate functions effectively as a TE generator, successfully converting ∆*T*_⊥_ between the planar heat source and the atmosphere into ∆*T*_∥_, thereby enabling a functional TE response.

One of the critical limitations of thin-film TE devices is that, even when a temperature difference is initially established, the thin platform rapidly reaches thermal equilibrium. As a result, Δ*T* tends to diminish or disappear over time. To evaluate whether the Δ*T* formed by the proposed dual-κ substrate could be stably maintained, we monitored the temporal evolution of *T*_s_ of the PDMS and Cu-PDMS regions. Figure 4D presents the *T*_s_ profiles of the two regions over time, starting from the moment the device was placed on a heat source at 66°C, as measured using an infrared (IR) camera. The surface temperatures rose rapidly and stabilized at 56.4°C (PDMS) and 64.6°C (Cu-PDMS) within ~100 s, thereby establishing a consistent Δ*T*_∥_ of around 8.2° ± 0.3°C across the device (see note S3, figs. S8 and S9, and table S1 for extended time traces and fitting results). This temperature difference was sustained throughout a prolonged measurement period of 250 s, demonstrating the superior thermal stability of our dual-κ substrate compared with conventional thin-film platforms. The infrared (IR) camera images taken at 20, 100, and 200 s (Fig. 4E) further support these observations. The thermal maps reveal a uniform temperature distribution within each region and show the formation of a temperature gradient near the junction. This confirms that the generated Δ*T*_∥_ is not confined to a localized hotspot but is spatially uniform and stable. These observations qualitatively agree with the FEA-predicted temperature distribution of a single-cell pT-TEG, capturing the same overall spatial trend (fig. S10 and see note S4 for simulation details).

To further exploit the flexibility of the PDMS substrate, we also evaluated the mechanical durability of the pT-TEGs under repeated deformation. As shown in Fig. 4F, after 500 cycles of bending at a radius of 3 mm, the output voltage decreased only marginally from 0.57 to 0.51 mV. Cross-sectional SEM images (fig. S11) confirm that all interfaces remained intact, with no evidence of cracking or delamination after bending. This mechanical robustness highlights the potential of the pT-TEG architecture for use in flexible and wearable electronic systems.

Overall, these results demonstrate that the dual-κ platform can effectively redirect heat flow to achieve in-plane TE generation from a vertical heat flux without requiring specialized transverse TE materials. This approach enables the full advantages of organic systems, including higher Seebeck coefficients, excellent mechanical flexibility, and scalable low-cost processing. A comparison of conventional transverse TE materials with the pT-TEG platform is provided in table S2.

### Thin-film pT-TEG module with scalable and wearable design

**Fig. 5.**
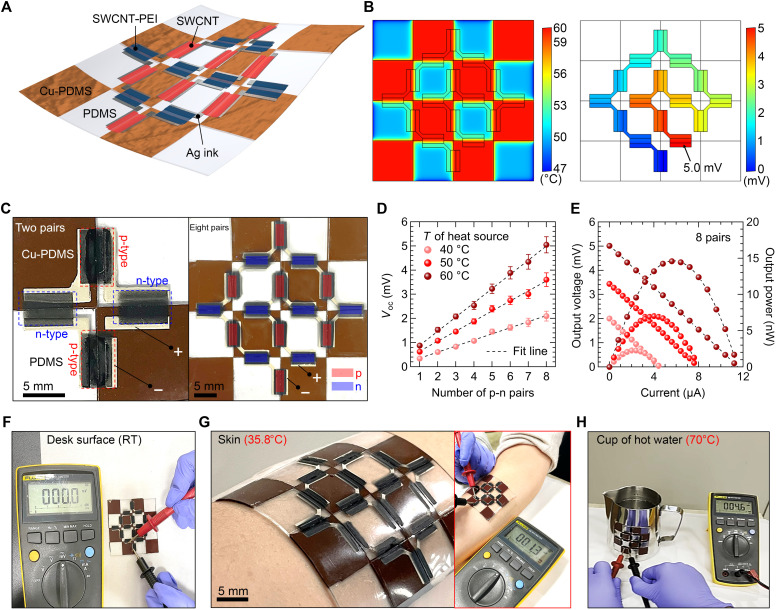
Demonstration of pT-TEG modules for wearable applications. (**A**) Schematic illustration of a soft and conformable pT-TEGs module comprising p- and n-type SWCNTs interconnected by Ag ink-based electrodes. (**B**) Simulation results showing the temperature distribution and output voltage of the eight-pair pT-TEG module. The simulation geometry is modeled to match the actual device dimensions, with 40 mm by 40 mm footprint and a total thickness of 400 μm. (**C**) Photographs of fabricated pT-TEG modules with two and eight p-n pairs. Scale bars, 5 mm. (**D**) Open-circuit voltage of the pT-TEG modules as a function of the number of p-n pairs, showing that *V*_oc_ increases proportionally with *T*_H_. Error bars indicate the SD from three independently fabricated modules. (**E**) Output power characteristics of the eight-pair pT-TEG module measured at different *T*_H_. (**F** to **H**) *V*_oc_ of the pT-TEG module measured on a plane surface (F), on human skin (scale bar, 5 mm) (G), and on the surface of a cup filled with hot water (H). RT, room temperature.

By optimizing our highly scalable fabrication process, we can easily fabricate pT-TEG modules by integrating multiple p- and n-type legs on expanded dual-κ substrates. N-type doping of the SWCNTs was achieved by applying a PEI solution onto the prepared SWCNTs (*15*), as confirmed by a red-shift in the Raman spectra (fig. S12) and changes in TE properties, i.e., the sign inversion of the Seebeck coefficients (fig. S13). The air stability of the PEI-doped SWCNT n-leg was also evaluated, showing minimal degradation in TE properties under air exposure (>100 hours; fig. S14). Figure 5A presents the schematic illustration of our rationally designed, soft, and conformable pT-TEG module. The module consists of 16 TE legs, comprising 8 p-type and 8 n-type SWCNT legs, interconnected by elastic Ag electrodes. These legs are positioned at the junctions of the Cu-PDMS and PDMS regions. This configuration enables unidirectional current flow: from the PDMS side to the Cu-PDMS side in n-type SWCNTs and in the reverse direction for p-type SWCNTs. As a result, the voltages from each leg can be additively combined. The operation of the module is visualized in FEA simulations of heat transfer and voltage distribution, as shown in Fig. 5B (see note S4 for details). The predicted theoretical output voltage of the 16-leg module is 5.0 mV at *T*_H_ of 60°C.

To demonstrate the scalability of the pT-TEGs, we fabricated integrated modules comprising one to eight pairs of p- and n- type SWCNT legs. Photographs of the two-pair and eight-pair square-shaped pT-TEG modules are shown in Fig. 5C. Crucially, these chessboard-patterned modules can be further extended into scalable and arbitrary configurations by assembling elemental units of p-n leg pairs, as illustrated in fig. S15. Using the two-pair module, we verified that the lateral temperature gradient Δ*T*_∥_ remains stable under bending, as confirmed by IR images (fig. S16). The open-circuit voltages (*V*_oc_) of the eight-pair modules, measured at various *T*_H_ ranging from 40° to 60°C, exhibit a highly linear relationship with the number of unit pairs (Fig. 5D and fig. S17). For instance, the eight-pair pT-TEG module generated 5.05 ± 0.33 mV at 60°C. Figure 5E shows the output voltage and power (*P*_out_) of the eight-pair module at different values of *T*_H_. The maximum *P*_out_ also increased proportionally with temperature, reaching 14.6 nW at 60°C. Both the maximum *P*_out_ and the internal resistance scale linearly with the number of p-n pairs (fig. S18), confirming the ideal series connection behavior of the modular architecture. The soft and conformable nature of our pT-TEGs, combined with their ability to control the direction of heat flux, make them highly suitable for wearable applications. To demonstrate this, we first placed the eight-pair module on a flat desk surface at room temperature to confirm its inactive state (Fig. 5F). When attached to human skin at a temperature of 35.8°C, the module generated an output voltage of 1.3 mV (Fig. 5G). Moreover, the pT-TEG showed reliable performance in everyday high-temperature environments; for example, when placed on a cup filled with hot water at 70°C, the device generated a stable output of 4.6 mV (Fig. 5H).

## DISCUSSION

This work presents a platform for wearable organic TEGs, capable of efficiently harvesting thermal energy from various dynamic surfaces owing to their unique ability to generate in-plane electrical power under longitudinal heat flux. The operation of the pT-TEGs is enabled by structurally mimicking transverse TE effects through the development of a dual-κ substrate, which combines two materials with distinct thermal conductivities. Through a combination of simulation and solution-based fabrication techniques, we demonstrated deformable and scalable pT-TEG modules featuring a chessboard layout, in which multiple p- and n-type legs are interconnected via printed elastic Ag electrodes. Furthermore, we showed that the modular architecture of the pT-TEG allows for its assembly into diverse configurations, enabling a wide range of application possibilities.

Looking ahead, several key directions emerge for advancing the dual-κ strategy. A primary route involves refining the geometry and κ ratio of the dual-κ substrate to enhance ∆*T*_∥_ under identical heating conditions. Lowering κ_low_, for example, through micro-porous or aerogel-polymer composites, is expected to further amplify ∆*T*_∥_. Introducing controlled porosity not only reduces the thermal conductivity of the low-κ region but also increases the interfacial thermal contact resistance (*R*_tc_) by decreasing the effective contact area and promoting micro-scale roughness at the κ_high_/κ_low_ boundary. Enhanced *R*_tc_ more effectively suppresses lateral heat spreading and preserves the induced ∆*T*_∥_. Once *R*_tc_ exceeds a critical threshold, steep lateral gradients can form over short distances, suggesting that microstructural tuning can enable compact, high-power density devices (see note S1 and fig. S3).

Expanding the range of active TE materials offers another promising avenue for improving performance. Because the dual-κ configuration reorients heat laterally without requiring structural transformation, various thin-film TE materials, such as conjugated polymers, CNT networks, or emerging 2D semiconductors, can operate in a pseudo-transverse mode. This universality was experimentally validated using commercially available PEDOT:PSS, where a pT-TEG fabricated on the dual-κ substrate exhibited a clear linear Δ*V*-Δ*T* response with an average Seebeck coefficient of 16.9 ± 2.2 μV K^−1^ (fig. S19). Integrating higher-*ZT* thin-film TE materials within the same architecture, together with optimized κ contrast and *R*_tc_ engineering, could substantially enhance power output and efficiency.

Last, process scalability and device miniaturization are essential for translating the pT-TEG concept into practical, high-density thermal energy harvesters. Advanced printing and microfabrication techniques could enable the integration of dense p-/n-type arrays into compact geometries, paving the way for thinner and more power-dense modules. Achieving this will require careful engineering of κ ratios and interfacial properties to sustain stable ∆*T*_∥_ and mechanical robustness as device dimensions shrink. Although further optimization is required to realize ultrathin configurations, the demonstrated scalability of the dual-κ substrate provides a strong foundation for future development.

Overall, the dual-κ substrate strategy introduced here addresses a fundamental limitation of conventional thin-film TEGs by enabling transverse-like TE generation directly within planar, 2D architectures without the need for geometric transformation. By enabling lateral voltage formation purely through heat-flow reorientation, this work establishes a practical and broadly applicable framework for pseudo-transverse operation in organic and 2D TE systems, thereby laying the groundwork for the next generation of flexible, scalable, and wearable energy-harvesting platforms.

## MATERIALS AND METHODS

### FEA simulations

Finite element simulations were conducted using the Heat Transfer in Solids and Electric Currents modules in COMSOL Multiphysics to model the temperature distribution in the dual-κ substrates and the TE behavior of the pT-TEGs. For the simulations shown in Fig. 2, simplified dual-κ substrate models without the full device stack were used to isolate and evaluate the heat-guiding mechanism and to optimize key geometrical parameters before device fabrication. For the device-level simulations, the geometry and material parameters were defined to match the actual pT-TEG structures, and material properties were taken from experimental measurements when available or from COMSOL defaults otherwise. To reproduce the experimental environment, a convective heat flux boundary condition was applied to top and edge surfaces. Unless otherwise specified, the heat transfer coefficient was set to *h* = 15 W m^−2^ K^−1^. Additional details of the simulations are provided in note S4.

### Preparation of dual-κ substrate

To fabricate a dual-κ substrate, thermally conductive ink was prepared by mixing stretchable silicone rubber, Cu nanopowder, and hexane. The base PDMS material (Sylgard 184, Dow) and curing agent were combined at a weight ratio of 10:1 and stirred mechanically for 30 min. After degassing the mixture, hexane was added as a solvent to dissolve the PDMS and mixed for an hour. Cu nanopowder was then added to the solution and mixed for 2 hours to obtain a homogeneous ink. Then, the prepared composite ink was poured into a customized Teflon mold and cured at room temperature for 20 min to evaporate the solvent, followed by partial curing in an oven at 60°C for an hour. After removing the half of the Cu-PDMS, pristine PDMS (10:1) was filled into the vacant area of the mold. After a final curing step, the dual-κ substrate was detached from the mold. The thickness of the substrate was 400 μm.

### Fabrication of pT-TEGs

The SWCNT solution (Korbon Co. Ltd.) in ethanol (1 wt %) was deposited by spray coating, followed by the thermal annealing at 70°C for 30 min. To make the n-type TE legs, a 1 wt % solution of PEI (Sigma-Aldrich) in ethanol was dropped onto the predeposited SWCNT film, followed by drying at 70°C for 10 min. For the contact electrodes and interconnection between TE legs, Ag elastic ink was deposited through a screen-printing mask, followed by curing in an oven at 100°C for an hour. The Ag elastic ink was prepared from the composite of Ag flakes and Ecoflex, following a previously reported method (*55*). In detail, Ecoflex 00-30 (Smooth-On Inc.) was used as the elastomer. Part A and Part B of the Ecoflex (1:1 weight ratio) were mixed using a mechanical stirrer for 30 min and dissolved in methyl isobutyl ketone. After forming a homogeneous binder solution, Ag flakes (DSF-500MWZ-S, Daejoo Electronic Materials Co. Ltd.) were added to obtain the Ag elastic ink.

### Characterization

SEM images were obtained using a JSM-7800F Prime field-emission scanning electron microscope. Raman spectra were acquired using a inVia Raman spectrometer (Renishaw) equipped with a 532-nm neodymium-doped yttrium aluminum garnet (Nd:YAG) laser. Thermal diffusivity and specific heat capacity were measured using an LFA-467 HyperFlash thermal analyzer (Netzsch) and a DSC 214 differential scanning calorimeter, respectively. To characterize the output performance of the pT-TEGs, the device was placed on a Peltier module as the heat source. Δ*T* and Δ*V* were measured using a Keithley 2700 multimeter with two T-type thermocouples and a Keithley 2182A nanovoltmeter, respectively (*56*). An IR camera (FLIR, A325sc) was used to continuously visualize Δ*T* during operation, whereas quantitative temperatures were measured using thermocouples (see note S5). Output power was measured under thermal equilibrium conditions. Mechanical properties, including bending and stretching, were characterized using a customized automated stretching system and software.

### Ethics statement

The study was approved by the Institutional Review Board (IRB) of Seoul National University (IRB no. 2507/004-026). The wearable device demonstration included in this manuscript was performed solely by the author as a self-experiment under this IRB approval, and no external participants or personal identifiable data were collected.

## Supplementary Materials

### This PDF file includes:

Supplementary Notes S1 to S5 Figs. S1 to S19 Tables S1 and S2 References
