# Supplementary material for: All-solution-processed scalable and wearable organic thermoelectrics by structurally mimicking transverse thermoelectric effects
Source: Sci Adv. 2026 Mar 18;12(12):eaea9094. doi: 10.1126/sciadv.aea9094 (PMC12998524; doi:10.1126/sciadv.aea9094)
Supplement: Supplementary file 1 — Supplementary Notes S1 to S5 Figs. S1 to S19 Tables S1 and S2 References [file sciadv.aea9094_sm.pdf]

Science Advances
AAAS

Supplementary Materials for

All-solution-processed scalable and wearable organic thermoelectrics by structurally mimicking transverse thermoelectric effects

Juhyung Park *et al.*

Corresponding author: Jeonghun Kwak, jkwak@snu.ac.kr

Sci. Adv. **12**, eaea9094 (2026)
DOI: 10.1126/sciadv.aea9094

This PDF file includes:

Supplementary Notes S1 to S5
Figs. S1 to S19
Tables S1 and S2
References

Note S1. Thermal-circuit and geometry design framework for dual-κ substrates

In a dual-κ substrate, a high-thermal-conductivity region (κ_{high}) and a low-thermal-conductivity (κ_{low}) region are placed adjacent to each other laterally. When the substrate is heated from below under a near-isothermal boundary, heat predominantly flows vertically within each region, resulting in different surface temperatures: a higher surface temperature $T_{s,h}$ on the high-κ side and a lower surface temperature $T_{s,c}$ on the low-κ side. This vertical heat-conduction asymmetry establishes a lateral surface temperature difference $\Delta T_{\parallel} = T_{s,h} - T_{s,c}$. In the absence of any lateral heat exchange, this vertical effect alone would define the surface temperature difference.

However, in practice, lateral thermal coupling between the two regions partially relaxes this temperature difference. To isolate this lateral redistribution mechanism, we introduce a thermal-resistor model that considers only in-plane heat conduction while treating the vertical temperature contrast as a fixed boundary condition. The lateral heat-flow path consists of serial conduction through the two regions, along with a finite thermal contact resistance $R_{tc} = 1/(h_c A)$ (K W^{-1}) (*53*), where h_c is the thermal contact conductance and A is the cross-sectional area. The total lateral thermal resistance is therefore, $R_{lat} = R_{high} + R_{tc} + R_{low}$, where $R_{high(low)} = L_{high(low)}/(\kappa_{high(low)}A)$, where $L_{high(low)}$ represent the effective lateral conduction lengths in the κ_{high} and κ_{low} regions, respectively. Assuming identical cross-sections and uniform top-surface convection, the retained lateral temperature contrast can be expressed relative to the contrast that would exist in the absence of any lateral heat exchange. We denote this idealized vertical-transport-limited contrast as $\Delta T_{\parallel}^{intrinsic}$, corresponding to the case $R_{tc} \to \infty$. Under these assumptions, the retained temperature contrast scales approximately as

$$\frac{\Delta T_{\parallel}(R_{tc})}{\Delta T_{\parallel}^{\mathrm{intrinsic}}} \approx \frac{R_{tc}}{R_{high} + R_{tc} + R_{low}} \tag{S1}$$

Therefore, increasing R_{tc} suppresses lateral heat exchange and preserves a larger fraction of the intrinsic κ-driven surface temperature difference originally generated by vertical thermal transport. In the limit $R_{tc} \to \infty$, the two regions become laterally thermally isolated and the intrinsic contrast is fully retained, whereas $R_{tc} = 0$ corresponds to perfect lateral thermal coupling and complete relaxation of $\Delta T_{\parallel}$.

While the scaling relation above highlights the role of R_{tc} in preserving the κ-driven surface contrast, the spatial extent over which this enhancement is expressed depends on the device geometry. In particular, the interplay between the lateral conducting length L_D and the thermal healing length ℓ governs how $\Delta T_{\parallel}$ manifests spatially. The thermal healing length ℓ represents the distance over which a lateral surface-temperature gradient decays due to the competition between in-plane thermal conduction and heat loss to the environment (*57*). For a thin substrate, this length scale can be estimated as

$$\ell \sim \sqrt{\frac{\kappa_{eff} t_{sub}}{h_{eff}}} \tag{S2}$$

where t_{sub} is the substrate thickness, κ_{eff} is the effective in-plane thermal conductivity of the dual-κ medium, and h_{eff} denotes the effective convective/radiative heat transfer coefficient to the surroundings (*58*). When the lateral conducting length is comparable to or smaller than the thermal healing length ($L_D \leq \ell$), the interface region dictates the surface temperature distribution, and increasing R_{tc} amplifies the observed $\Delta T_{\parallel}$ across the entire device. In contrast, when the lateral conducting length greatly exceeds the healing length ($L_D >> \ell$), the global temperature drop remains primarily governed by the κ-contrast, while R_{tc} only sharpens and extends the local

interfacial gradient without affecting the far-field temperature difference dictated by the vertical κ-contrast. Thus, R_{tc} does not create the thermal contrast itself; rather, it controls how effectively the intrinsic κ-induced contrast is retained over a finite lateral distance. In practical device design, these results imply that the lateral geometry and thermal contact resistance should be co-optimized. In particular, selecting an electrode spacing L_E on the order of the thermal healing length ℓ maximizes the expression of the κ-induced temperature difference, while engineering sufficient thermal contact resistance R_{tc} suppresses in-plane thermal relaxation. Within fabrication and packaging constraints, targeting $L_E \sim \ell$ and minimizing unintended thermal bridging at the interface provide a general design guideline for maximizing the pseudo-transverse thermoelectric response in dual-κ substrates.

To verify the physical trends predicted by this model, we also performed steady-state FEA simulations (fig. S3). The numerical results are fully consistent with the analytical picture: (i) increasing thermal contact resistance preserves κ-induced contrast over longer distances, and (ii) increasing κ contrast increases the intrinsic vertical temperature asymmetry. Details of the simulation framework and boundary conditions are presented in Note S4.

Note S2. Interfacial fracture energy of the Cu–PDMS/PDMS joint

We quantified the interfacial fracture energy (Γ) between Cu–PDMS and PDMS using a Rivlin–Thomas–type protocol with paired unnotched/notched tensile samples as shown in fig. S6 (*59*, *60*). Experiments were performed on an Instron 3367 (500 N load cell; peel rate 50 mm min^{-1}). For each pair, the unnotched sample was pulled in tension to obtain the force-clamp distance curve $F(L)$. The notched sample (interfacial notch at the junction) was tested under the same setup; the critical clamp distance L_c at which the notch transitions to a running interfacial crack (onset of the sharp post-peak force drops). The mechanical work done to the unnotched specimen up to L_c is

$$U(L_C) = \int_0^{L_c} F(L)dL \qquad (S3)$$

The interfacial fracture energy is then obtained by normalizing the work with the initial width a_0 and thickness b_0 of the notched specimen:

$$\Gamma = \frac{U(L_c)}{a_0 b_0} \qquad (S4)$$

For our samples, the initial width and thickness were a_0 = 16.3 mm and b_0 = 0.49 mm, respectively. The critical clamp distance was L_c = 6.594 mm, and integrating the unnotched *F*–L curve up to L_c gave $U(L_c)$ = 21.55 N mm. Using equation (S4), this yields an Γ ≈ 2700 J m^{-2}. This value is substantially higher than typical adhesion energies reported for untreated polymer interfaces (10–100 J m^{-2}) (*54*) or even chemically treated polymer/metal junctions (100–500 J m^{-2}). (*61*) Values exceeding 1000 J m^{-2} are generally regards as indicative of strong interfacial bonding. (*62*) Thus, the Cu–PDMS and PDMS joint exhibits robust adhesion that resists crack propagation under routine handling and bending.

Note S3. Transient analysis of surface temperature response

The heating and relaxation dynamics of the Cu–PDMS and PDMS regions were analyzed using a step-wise bi-exponential model:

$$T(t) = \begin{cases} T_0, & t < t_{\mathrm{on}} \\ T_0 + (T_{\mathrm{ss}} - T_0)\left[1 - \left(a e^{-\frac{t}{\tau_{1\mathrm{h}}}} + (1-a) e^{-\frac{t}{\tau_{2\mathrm{h}}}}\right)\right], & t_{\mathrm{on}} \le t < t_{\mathrm{off}} \\ T_0 + (T(t_{\mathrm{off}}) - T_0)\left[a e^{-\frac{t - t_{\mathrm{off}}}{\tau_{1\mathrm{r}}}} + (1-a) e^{-\frac{t - t_{\mathrm{off}}}{\tau_{2\mathrm{r}}}}\right], & t \ge t_{\mathrm{off}} \end{cases} \quad \text{(S5)}$$

where T_0 denotes the initial temperature, T_{ss} the steady-state surface temperature during heating, $t_{on,}$ and t_{off} the switching times for heating-on and heating-off, *a* the weighting factor between fast (τ_1) and slow (τ_2) processes, and τ_{1h}, τ_{2h}, τ_{1r}, τ_{2r} the characteristic time constants for heating and relaxation, respectively. As shown in fig. S8, both Cu–PDMS and pristine PDMS exhibit a rapid rise upon heating and a subsequent thermal relaxation once the Peltier is switched off, which are well described by the above model. Importantly, the surface temperatures remain steady up to the switching time t_{off} under a constant heat source at T_H = 66 °C, with no evidence of thermal drift. The temperature difference stabilized at $\Delta T_{\parallel} \approx 8.2$ °C determined from the last 10% of the raw data points. The extracted parameters (Table S1) indicate two distinct thermal timescales: a fast response (τ_1) associated with interfacial heating and a slower component (τ_2) reflecting bulk thermal diffusion. Compared to pristine PDMS, Cu–PDMS shows a higher steady-state temperature and stabilizes faster, highlighting the beneficial role of conductive filler. Longer-duration monitoring at a lower heat-source setting (T_H = 60 °C, up to ~2500 s) likewise demonstrates a stable $\Delta T_{\parallel}$ without drift; see fig. S9. Overall, this analysis demonstrates that the dual-κ substrate not only establishes a temperature difference quickly but also maintains it robustly over extended heating periods.

Note S4. FEA simulation of the pT-TEG devices

A steady-state FEA simulation was performed to evaluate the heat transfer characteristics of the pT-TEG devices and to compare the resulting temperature distribution with experimental results. A multilayer 3D geometry was constructed, consisting of the Peltier module, the dual-κ substrate composed of Cu–PDMS (κ_{high} = 0.88 W m^{-1} K^{-1}) and pristine PDMS (κ_{low} = 0.15 W m^{-1} K^{-1}), an SWCNT thermoelectric layer, and flexible Ag-composite electrodes. All geometrical parameters and materials properties (including thermal conductivity, electrical conductivity) were set to match the fabricated devices. In the experiment, heating is supplied by an external Peltier module positioned beneath the substrate. To emulate this configuration, a solid Ag block was placed directly below the substrate in the model. The bottom surface of this Ag block was fixed to the experimentally measured heater temperature for each device configuration (i.e., T_H = 60 or 66 °C), while no other surface was held at a fixed temperature. Convective heat transfer (h = 15 W m^{-2} K^{-1}) and surface-to-ambient radiation were applied to the top and lateral surfaces, using material-specific emissivity for the electrodes, SWCNT, Cu–PDMS, and PDMS regions. Interfacial heat transport across the κ-contrast boundary was modeled using an aerial thermal contact resistance of R'_{tc} = 1 × 10^{-4} m^2 K W^{-1}, representative of polymer-polymer and polymer-composite interfaces. This value falls within the literature-reported range (10^{-5}–10^{-3} m^2 K W^{-1}) (*63*–*65*) and its influence on $\Delta T_{\parallel}$ is evaluated in fig. S3. A representative steady-state temperature distribution for a single-cell pT-TEG at T_H = 66 °C is shown in fig. S10. The simulations for 8 pairs pT-TEG modules presented in the main text (Fig. 5B) were conducted under the same modeling assumptions and boundary conditions with the heat-source temperature set to T_H = 60 °C to match the experimental measurement. For the 8-pair module, the Electrical Currents interface was also enabled, grounding one electrode and leaving the other floating to obtain the open-circuit voltage.

Note S5. Use of IR thermography and thermocouple measurements for temperature analysis

An infrared camera was employed in this study to visualize the spatial temperature distribution and the temporal stability of the in-plane temperature gradient during device operation. The IR images shown in Fig. 4E provide a qualitative view of the temperature distribution across the Cu–PDMS and PDMS regions and confirm the formation of a stable in-plane temperature gradient near the κ-contrast interface. However, IR camera–based temperature measurements are influenced by experimental factors such as the finite spatial resolution of the detector relative to the device feature size and emissivity differences between constituent materials. In the present pT-TEG devices, the emissivity contrast between the Cu–PDMS and pristine PDMS regions, together with spatial averaging inherent to IR imaging, can lead to smoothing of the apparent temperature gradient near the interface. For this reason, thermocouple measurements were used as the primary source of quantitative temperature values for device performance analysis and for validation of FEA simulations. As shown in Fig. S10, the in-plane temperature profiles measured by thermocouples show good agreement with the FEA-predicted temperature distribution under identical heating conditions, confirming that the thermal model accurately captures the experimentally observed temperature difference.

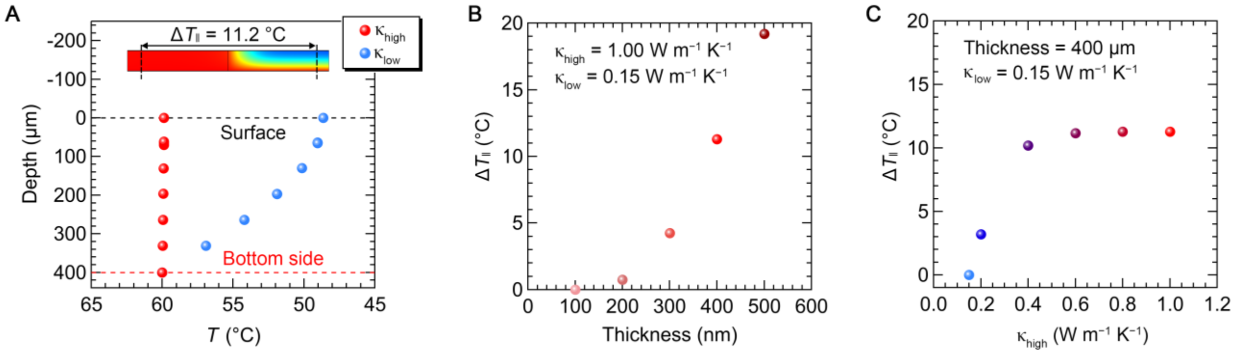

Fig. S1.

Finite element analysis (FEA) simulations of heat transfer on the dual-κ substrate. (**A**) Depth profile of the of the dual-κ substrates, showing a substantial temperature decrease from the bottom to the surface in the κ_{low} region, while the temperature remains nearly constant in the κ_{high} region. The resulting in-plane temperature gradient ($\Delta T_{\parallel}$) is 11.2 °C. (**B**) The $\Delta T_{\parallel}$ of the dual-κ substrate plotted as a function of the κ_{high}, and (**C**) as a function of substrate thickness. In both cases, κ_{low} is fixed at 0.15 W m^{-1} K^{-1}.

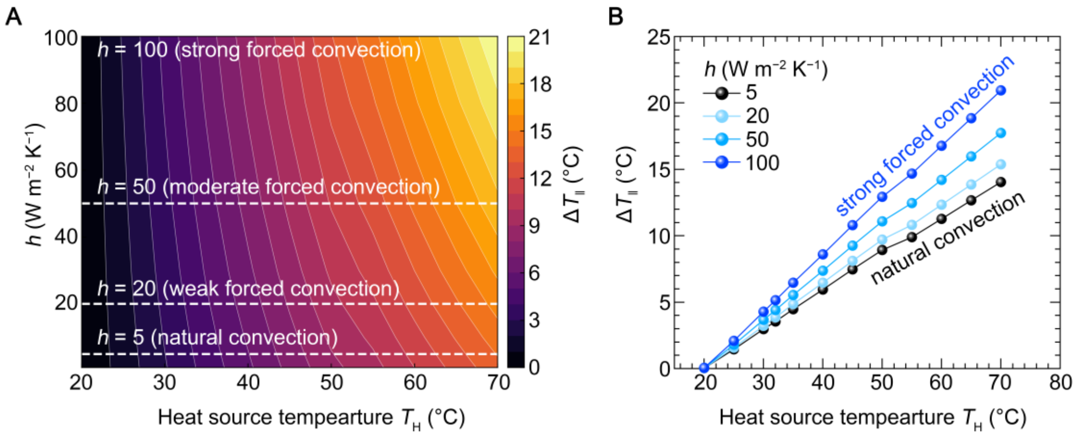

Fig. S2.

Effects of heat transfer coefficient h and heat source temperature T_H on in-plane temperature gradient $\Delta T_{\parallel}$. (**A)** Contour plot of $\Delta T_{\parallel}$ as a function of the h and the heat source temperature (T_H). The dashed white lines indicate representative cases: h = 5 W m^{-2} K^{-1} (natural convection), h = 20 W m^{-2} K^{-1} (weak forced convection), h = 50 W m^{-2} K^{-1} (moderate forced convection), and h = 100 W m^{-2} K^{-1} (strong forced convection). (**B**) Line-cut profiles of $\Delta T_{\parallel}$ versus T_H at these h values, showing that $\Delta T_{\parallel}$ monotonically increases with both h and T_H.

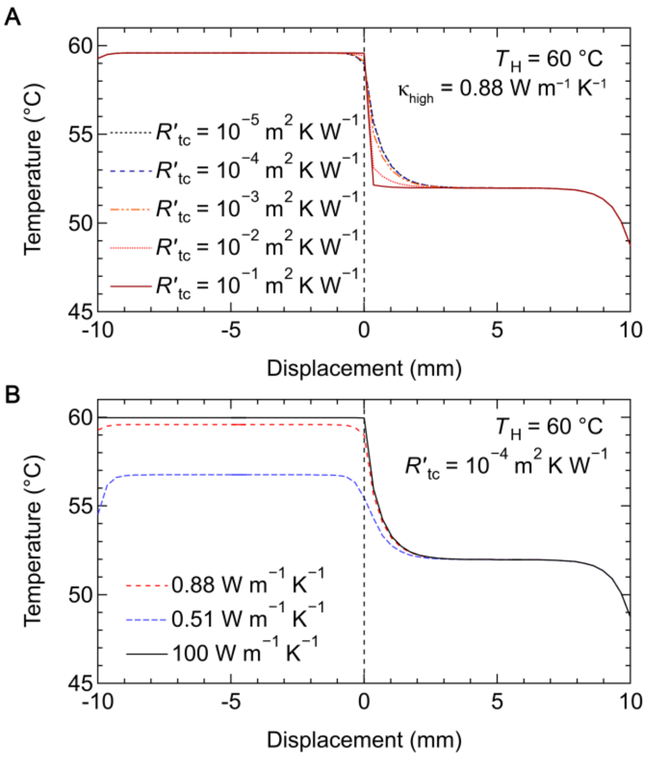

Fig. S3.

FEA simulations validating the dual-κ model. (A) Increasing areal thermal contact resistance R'_{tc} suppresses lateral heat spreading and preserves κ-induced contrast. (B) Increasing κ_{high} increases the intrinsic vertical temperature asymmetry. In both cases, far-field temperatures converge, consistent with the analytical prediction that vertical κ contrast generates the temperature gradient, while R'_{tc} governs its lateral retention. R'_{tc} is the area-normalized thermal contact resistance, defined as $R'_{tc} = R_{tc}/A$.

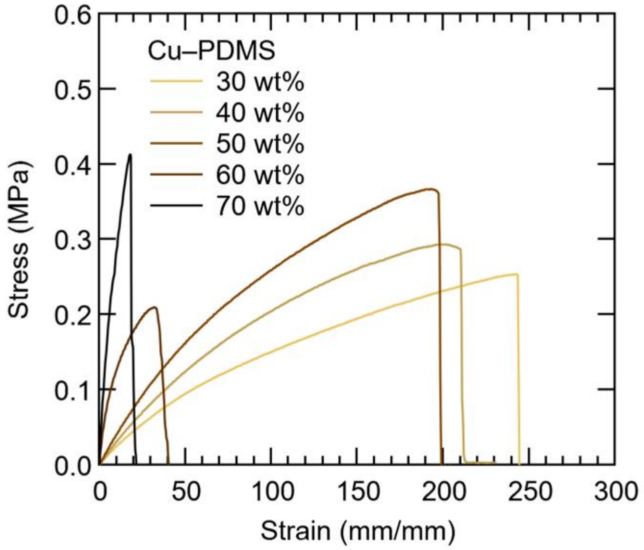

Fig. S4.

Mechanical properties of Cu-PDMS composites. Stress-strain curves for Cu-PDMS composites with different weight ratios of Cu microparticles are presented. The modulus increases as a function of the Cu microparticle weight ratio, while still maintaining elastic deformation.

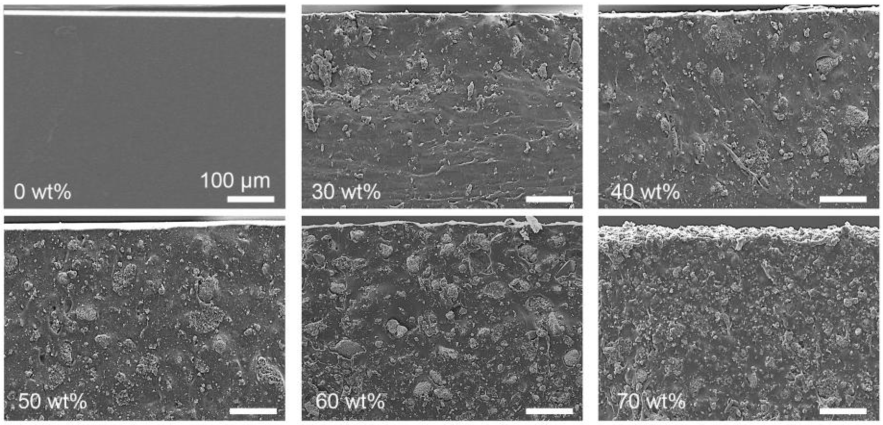

Fig. S5.

SEM images of PDMS and Cu-PDMS composite. Cross-sectional SEM images show Cu-PDMS composites with different weight ratios of Cu microparticles. The scale bars are 100 µm.

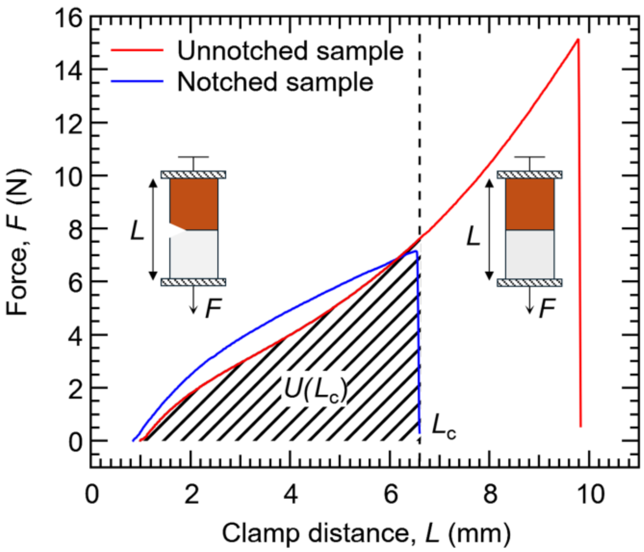

Fig. S6.

Experimental determination of the interfacial fracture energy. *F*–*L* curve curves of the unnotched (red) and notched (blue) to determine the fracture energy of Cu–PDMS/PDMS interface. An interfacial starter notch (~1 mm, sharp tip) was introduced along the Cu–PDMS/PDMS junction. The vertical dashed line indicates the critical clamp distance L_c taken from the notched test, where the interfacial notch begins to run. The hatched area under the red curve up to L_c represents the mechanical work delivered to the unnotched strip; normalizing this work by the initial width and thickness of the notched sample gives the interfacial fracture energy Γ. Insets illustrate the two test configurations and loading directions.

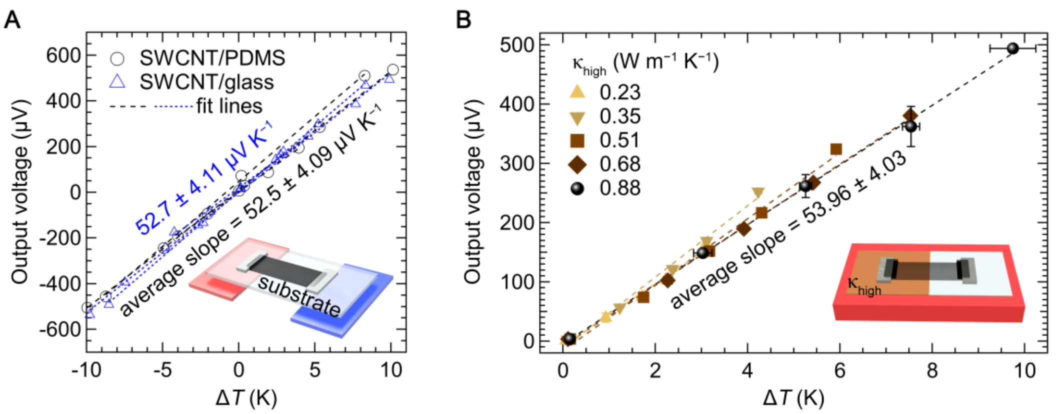

Fig. S7.

Output voltage of SWCNT as a function of temperature difference under different setups and substrates. (A) Output voltage of a p-type SWCNT film on a PDMS substrate measured using the conventional two-sided heating geometry (hot and cold Peltier modules imposing ΔT across the device). The extracted Seebeck slopes are 52.5 ± 4.09 μV K^{-1} (SWCNT/PDMS) and 52.7 ± 4.11 μV K^{-1} (SWCNT/glass). (**B**) Output voltage of SWCNT films supported on dual-κ substrate measured with a single heat source, where ΔT is induced laterally through κ contrast. Devices with different κ_{high} (0.23–0.88 W m^{-1} K^{-1}) show nearly identical slopes (average = 53.96 ± 4.03 μV K^{-1}), which is comparable to the intrinsic Seebeck coefficient of the SWCNT film within the experimental uncertainty. Error bars represent the standard deviation obtained from five independent devices.

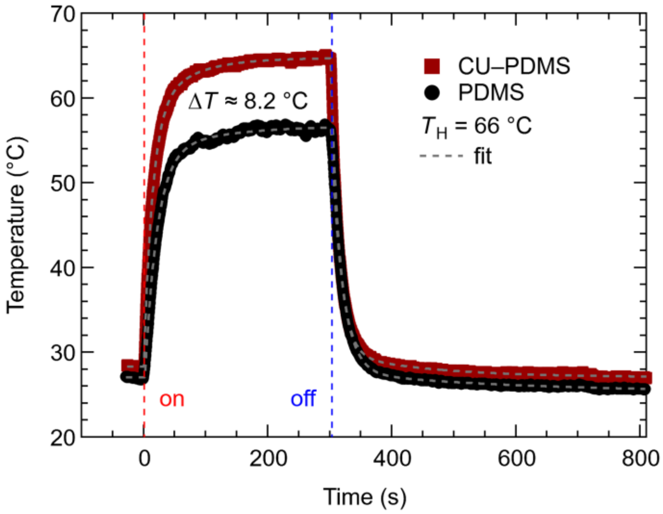

Fig. S8.

Surface temperature transients of Cu–PDMS and PDMS under heating-relaxation cycles. The surface temperatures of Cu–PDMS and pristine PDMS were monitored under a constant heat source at T_H = 66 °C using an infrared (IR) camera. The heating (on) and relaxation (off) switching times are indicated by the red and blue dashed lines, respectively. The experimental data (symbols) are well described by stepwise bi-exponential fits (grey dashed lines), capturing both the heating phase and the subsequent relaxation. The temperature difference stabilizes at $\Delta T_{\parallel} \approx 8.2 \pm 0.3$ °C, which is maintained robustly until t_{off} with no evidence of thermal drift.

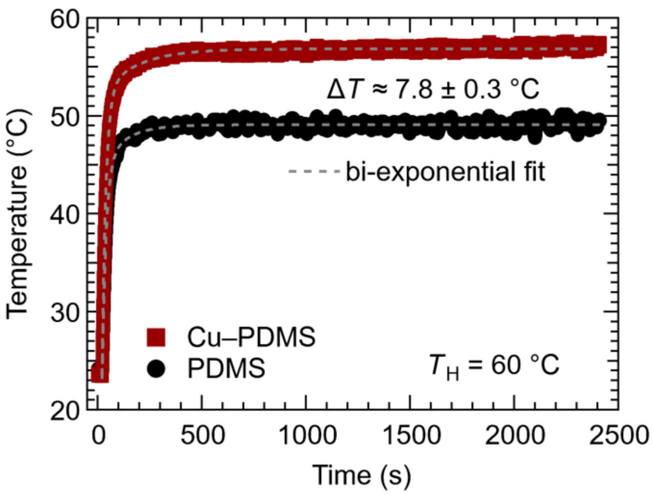

Fig. S9.

Long-term surface temperature stability of Cu–PDMS and PDMS. The surface temperatures of Cu–PDMS and pristine PDMS were monitored under a constant heat source at T_H = 60 °C using an infrared (IR) camera. Both samples rapidly reach steady state, after which the temperature difference stabilizes at $\Delta T_{\parallel} \approx 7.8 \pm 0.3$ °C. This stable temperature difference is maintained for more than 2000 s with no evidence of thermal drift.

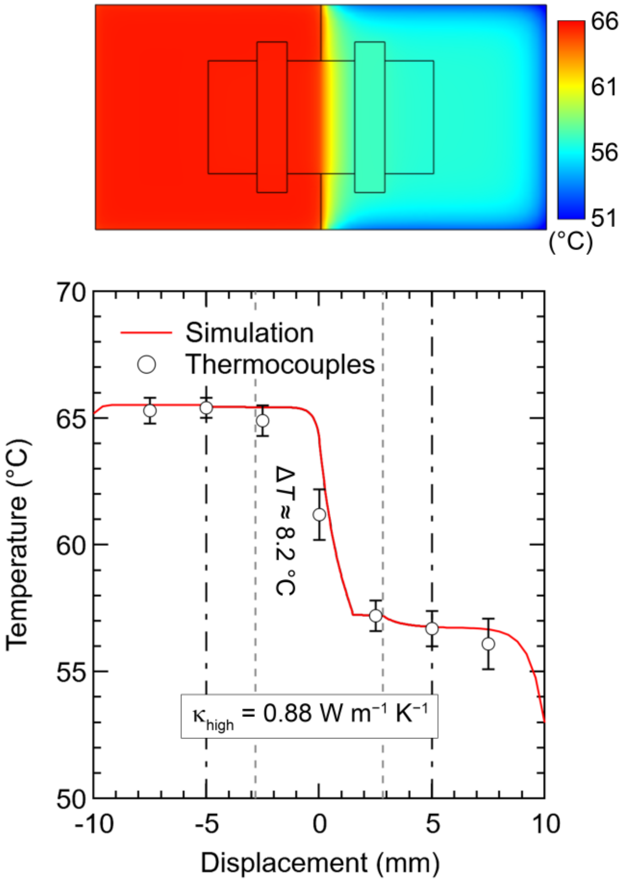

Fig. S10.

Thermal analysis of a single-cell pT-TEG device. (Top) FEA-simulated temperature distribution across the dual-κ substrate under bottom-side heating (T_H = 66 °C). (Bottom) Comparison between in-plane temperature profiles obtained from FEA simulations and thermocouple measurements, demonstrating good agreement with an in-plane temperature drop of $\Delta T_{\parallel} \approx 8.2$ °C across the Cu–PDMS (κ_{high} = 0.88 W m^{-1} K^{-1}) and PDMS (κ_{low} = 0.15 W m^{-1} K^{-1}) interface. Data points represent the mean ± standard deviation from five independently fabricated devices.

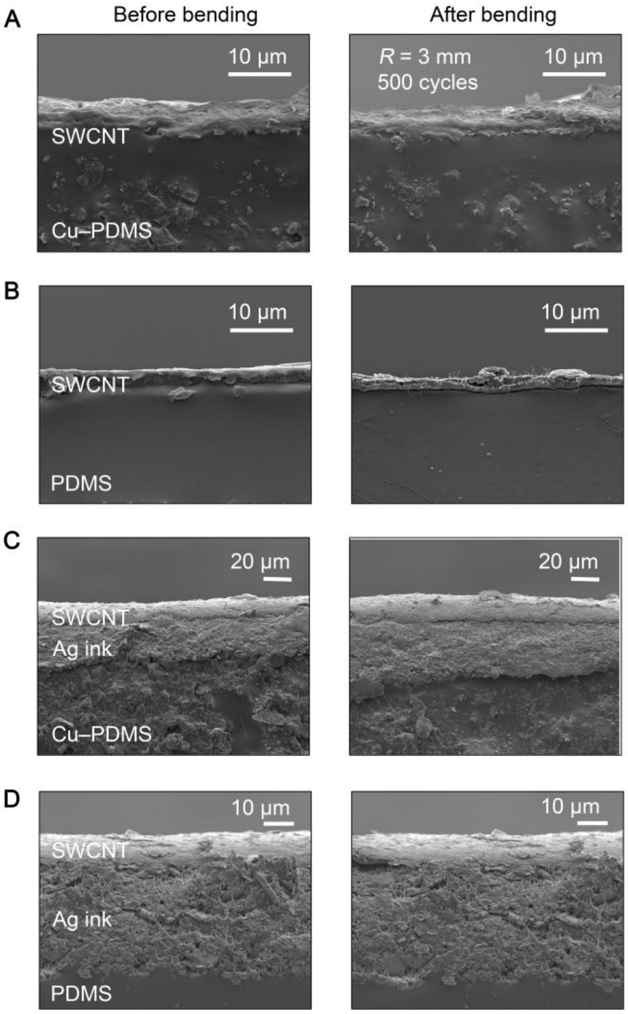

Fig. S11.

Cross-sectional SEM images of the substrate interfaces before and after bending. (A) SWCNT/ Cu–PDMS, (B) SWCNT/PDMS, (C) SWCNT/Ag ink/Cu–PDMS, and (D) SWCNT/Ag ink/PDMS structures before and after 500 bending cycles (R = 3 mm). All interfaces remained intact without visible cracks, delamination, or void formation after repeated bending, demonstrating the strong adhesion between the substrate, active and electrode layers. These results confirm that the electrical stability of the device is not affected by interfacial degradation, but is maintained by the mechanical integrity of the multilayer structure.

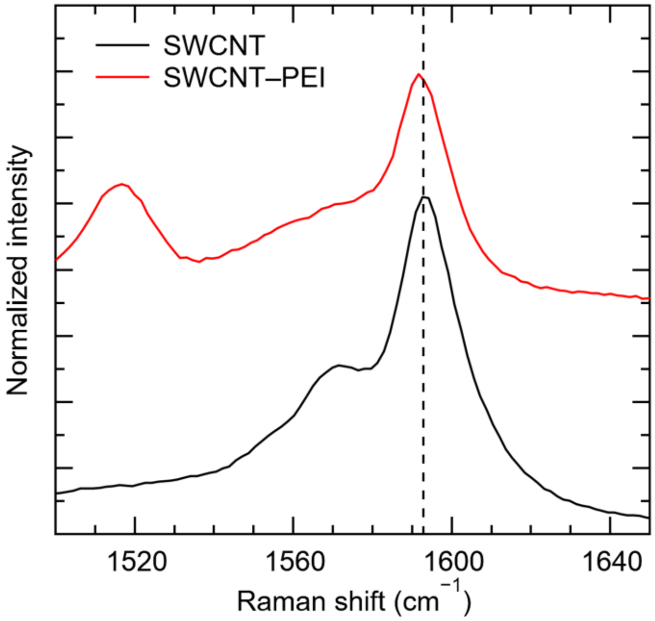

Fig. S12.

Normalized Raman spectra of SWCNT, and SWCNT–PEI films. The G-band peak for the SWCNT is observed at 1592.6 cm^{-1}. After treatment with polyethylenimine (PEI), the peak shifts to 1591.6 cm^{-1}. This downshift in the Raman frequency indicates charge transfer from PEI to SWCNT, suggesting n-doping of the SWCNT.

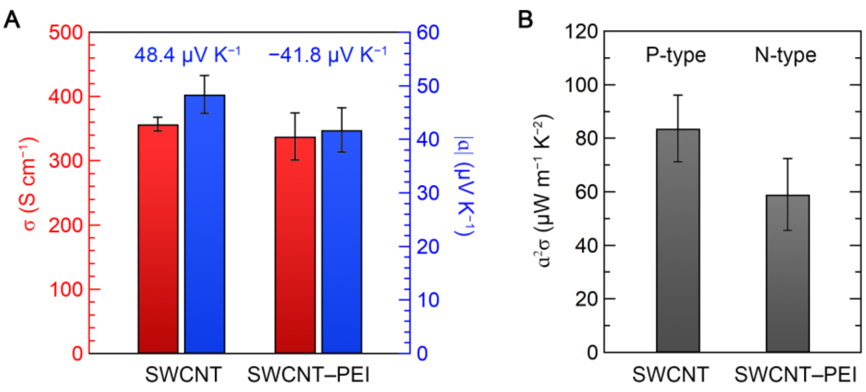

Fig. S13.

Thermoelectric properties of SWCNT and SWCNT–PEI films. (**A**) Electrical conductivity σ, Seebeck coefficient α and (**B**) power factor $\alpha^2\sigma$ for both SWCNT and SWCNT–PEI films.

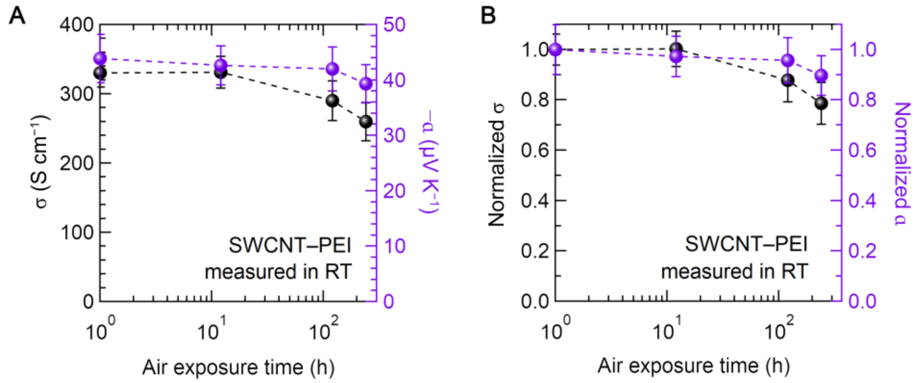

Fig. S14.

Ambient stability of PEI-doped SWCNT films. (**A**) Electrical conductivity σ, (black) and Seebeck coefficient α, (purple) as a function of air exposure time measured at room temperature. (**B**) Normalized σ and α, showing retention of ~100% after 12 h, ~ 88% after 120 h, and ~79% after 240 h of air exposure, indicating minimal degradation of TE properties over 100 h.

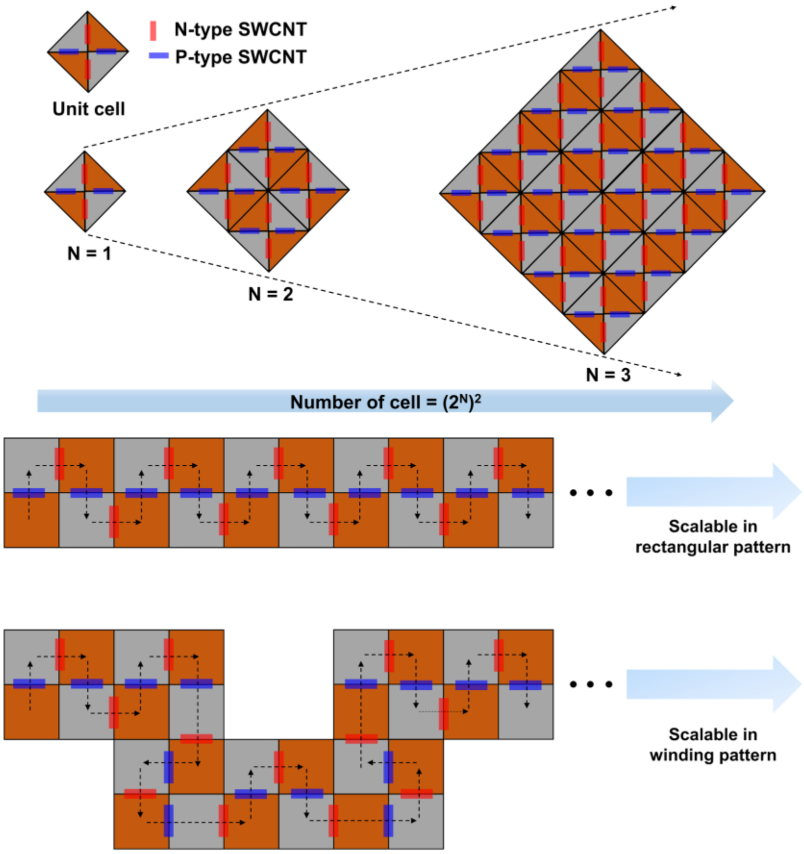

Fig. S15.

Scalable and versatile configurations of pT-TEG modules. The modules can be scaled by forming a chessboard-shaped square through repetition of N unit cells, each consisting of two p-type and two n-type SWCNT legs and assembling the chessboard-shaped units into arbitrary layouts, such as rectangular or meandering patterns, thereby demonstrating the structural adaptability of the design.

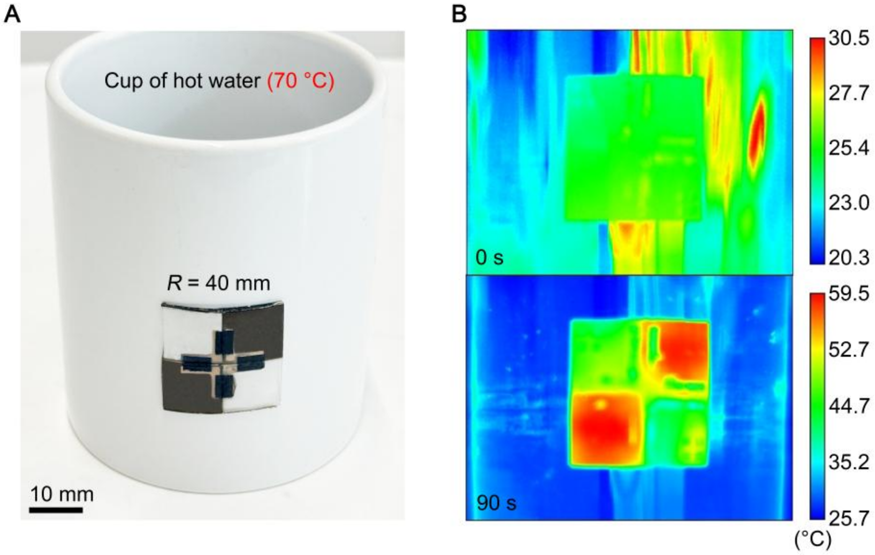

Fig. S16.

Stability of Δ$T_{\parallel}$ in the bent state. (A) Photograph of a supplementary thermoelectric module attached to a cup containing hot water (70 °C, R = 40 mm) under a bent condition. (B) Infrared thermal images of the module at 0 s and 90 s, demonstrating the stable temperature gradient during bending.

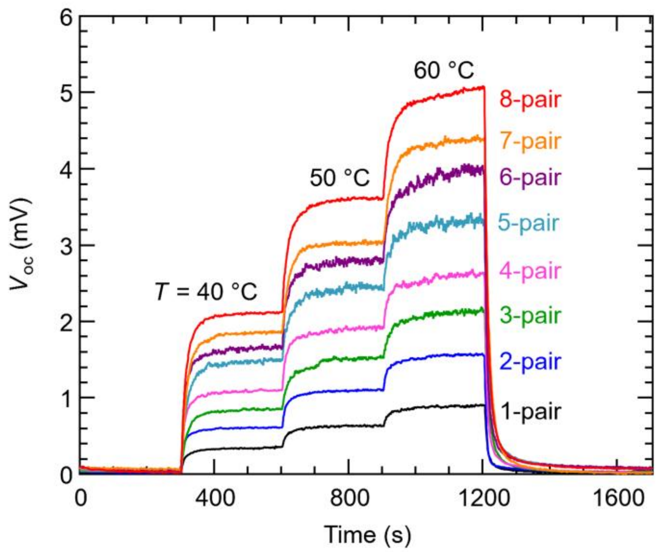

Fig. S17.

Open circuit voltage as a function of temperature and the number of modules. The V_{oc} values of the modules were measured over time, with heat source temperature ranging from 40 °C to 60 °C.

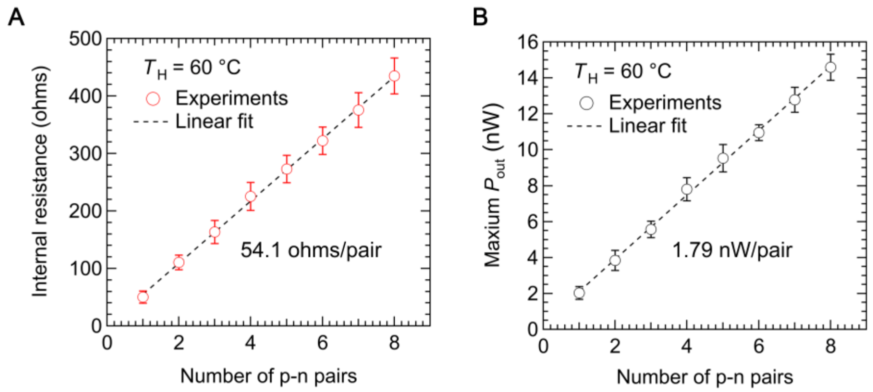

Fig. S18.

Internal resistance and maximum output power (P_{out}) of pT-TEG module as a function of the number of p–n pairs under T_H = 60 °C.
(A) Internal resistance increases linearly at approximately 54.1 ohms per pair, and (B) maximum P_{out} scales linearly at approximately 1.79 nW per pair, confirming ideal series behavior and negligible interfacial loss. Error bars represent standard deviations from three devices.

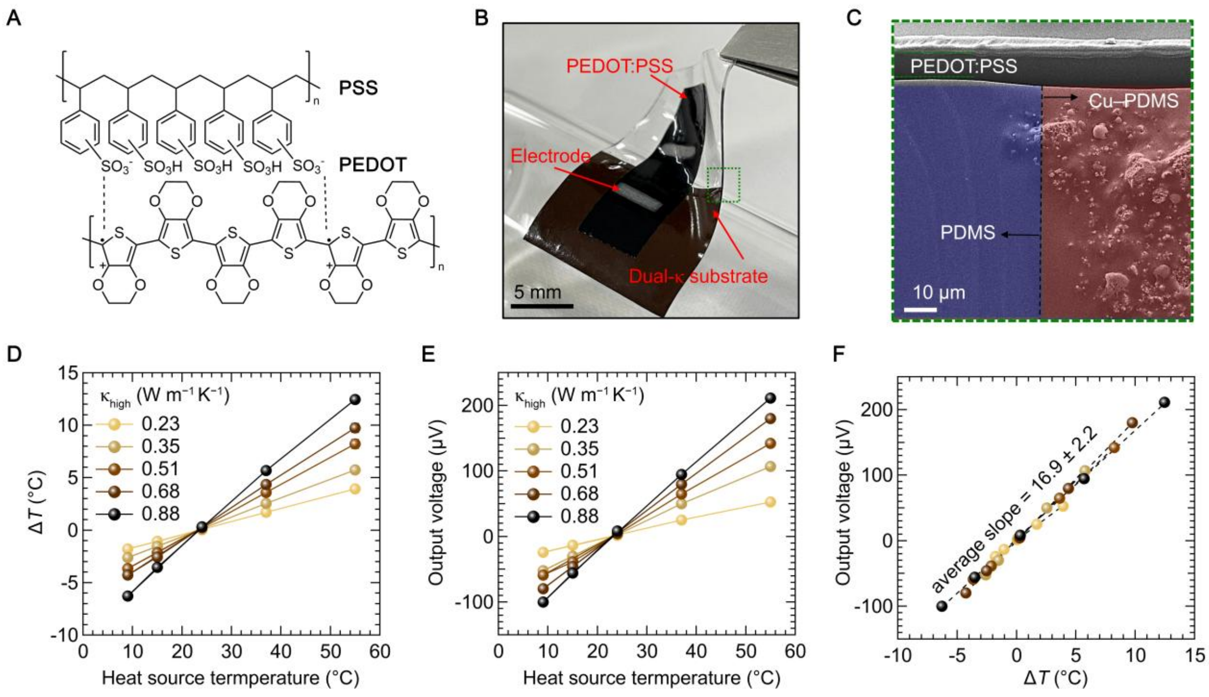

Fig. S19.

Demonstration of pT-TEG using commercially available PEDOT:PSS. (A) Chemical structure of PEDOT:PSS, (B) Photograph of the PEDOT:PSS-based pT-TEG device fabricated on a dual-κ substrate. (C) Cross-sectional SEM image showing the PEDOT:PSS layer on the dual-κ substrate (PDMS and Cu–PDMS). (D) Temperature difference across the PEDOT:PSS film as a function of the heat source temperature for substrates with different κ_{high} values. (E) Corresponding output voltage of the PEDOT:PSS pT-TEG device under the same conditions. (F) Linear relationship between ΔT and output voltage, yielding an average slope of 16.9 ± 2.2 μV K^{-1}, which is consistent with previously reported values for PEDOT:PSS (26).

Table S1.

Stepwise bi-exponential fitting parameters for surface temperature profiles of Cu–PDMS and PDMS. Parameters extracted from the stepwise bi-exponential fits shown in fig. S8. R^2 values indicate the goodness of fit for each regime.

Sample	T_{ss} (°C)	T_0 (°C)	a	τ_{1h} (s)	τ_{2h} (s)	τ_{1r} (s)	τ_{2r} (s)	R^2
PDMS	56.7	27.1	0.88	19.3	106.7	18.1	268.6	0.999
Cu–PDMS	64.7	28.3	0.81	13.7	62.8	13.6	145.3	0.999

Table S2.

Comparison of the pT-TEG with transverse thermoelectric materials. The table summarizes transverse thermopower, mechanical flexibility, processing temperature, and fabrication cost/scalability for this work and previously reported transverse thermoelectric platforms, including anomalous Nernst effect (ANE) and spin Seebeck effect (SSE) systems.

Materials	Mechanism	α (μV K^{-1})	Flexibility	Processing temperature (°C)	Cost / Process**	Reference
SWCNT (this work)	Seebeck (pT-TEGs)	~53*	Flexible	<100	Low (screen printing, solution)	This work
Mn_3Sn single crystal	ANE	0.35	Rigid	1080	High (Bridgman, low-throughput)	(*66*)
Co_2MnGa single crystal	ANE	6	Rigid	1250	High (arc/induction + slow cool)	(*67*)
Fe3Pt/ MgO	ANE	2	Rigid	600–900	High (UHV MBE)	(*68*)
Co/Pt	ANE	1.0	Rigid	Room Temp.	Medium (HV sputter + e-beam)	(*69*)
$Fe_{81}Al_{19}$	ANE	3.4	Flexible	Room Temp.	Medium (magnetron sputter, scalable)	(*70*)
Fe_3GeTe_2	ANE	0	Rigid	-	High (CVT + exfoliation)	(*40*)
Fe_3Sn polycrystal bar	ANE	2.9	Rigid	805	High (arc melt + sealed anneal)	(*41*)
Fe_3Ga	ANE	4	Rigid	500	Medium (sputter + anneal)	(*45*)
Fe_3Al	ANE	2	Rigid	450	High (UHV MBE + anneal)	(*45*)
YMn_6Sn_6 single crystal	ANE	2.0	Rigid	1000	High (Sn self-flux)	(*48*)
$Ni_{81}Fe_{19}$ / Pt wire	SSE	0.022	Rigid	-	Medium (HV evaporation + sputter)	(*44*)

*the Seebeck response measured under a transverse geometry (not intrinsic ANE/SSE).
**Abbreviations in Process: UHV—ultrahigh vacuum. MBE—molecular beam epitaxy. HV—high vacuum. CVT—chemical vapor transport.

REFERENCES

1. M. Lin, H. Hu, S. Zhou, S. Xu, Soft wearable devices for deep-tissue sensing. *Nat. Rev. Mater.* **7**, 850–869 (2022).

2. S. H. Kim, A. Basir, R. Avila, J. Lim, S. W. Hong, G. Choe, J. H. Shin, J. H. Hwang, S. Y. Park, J. Joo, C. Lee, J. Choi, B. Lee, K.-S. Choi, S. Jung, T.-i. Kim, H. Yoo, Y. H. Jung, Strain-invariant stretchable radio-frequency electronics. *Nature* **629**, 1047–1054 (2024).

3. W. Huang, Q. Ding, H. Wang, Z. Wu, Y. Luo, W. Shi, L. Yang, Y. Liang, C. Liu, J. Wu, Design of stretchable and self-powered sensing device for portable and remote trace biomarkers detection. *Nat. Commun.* **14**, 5221 (2023).

4. S. S. Kwak, H.-J. Yoon, S.-W. Kim, Textile-based triboelectric nanogenerators for self-powered wearable electronics. *Adv. Funct. Mater.* **29**, 1804533 (2019).

5. J. Min, S. Demchyshyn, J. R. Sempionatto, Y. Song, B. Hailegnaw, C. Xu, Y. Yang, S. Solomon, C. Putz, L. E. Lehner, J. F. Schwarz, C. Schwarzinger, M. C. Scharber, E. Shirzaei Sani, M. Kaltenbrunner, W. Gao, An autonomous wearable biosensor powered by a perovskite solar cell. *Nat. Electron.* **6**, 630–641 (2023).

6. Y. Wang, W. Zhu, Y. Deng, B. Fu, P. Zhu, Y. Yu, J. Li, J. Guo, Self-powered wearable pressure sensing system for continuous healthcare monitoring enabled by flexible thin-film thermoelectric generator. *Nano Energy* **73**, 104773 (2020).

7. H. Cho, D. Jang, J. Yoon, Y.-S. Ryu, B. Lee, B. Lee, S. Chung, Y. Hong, Milliwatt-scale body-heat harvesting using stretchable thermoelectric generators for fully untethered, self-sustainable wearables. *ACS Energy Lett.* **8**, 2585–2594 (2023).

8. S. Hwang, D. Jang, B. Lee, Y.-S. Ryu, J. Kwak, H. Kim, S. Chung, All direct ink writing of 3D compliant carbon thermoelectric generators for high-energy conversion efficiency. *Adv. Energy Mater.* **13**, 2204171 (2023).

9. B. Lee, H. Cho, K. T. Park, J.-S. Kim, M. Park, H. Kim, Y. Hong, S. Chung, High-performance compliant thermoelectric generators with magnetically self-assembled soft heat conductors for self-powered wearable electronics. *Nat. Commun.* **11**, 5948 (2020).

10. E. W. Zaia, M. P. Gordon, P. Yuan, J. J. Urban, Progress and perspective: Soft thermoelectric materials for wearable and internet-of-things applications. *Adv. Electron. Mater.* **5**, 1800823 (2019).

11. Y. Jia, Q. Jiang, H. Sun, P. Liu, D. Hu, Y. Pei, W. Liu, X. Crispin, S. Fabiano, Y. Ma, Y. Cao, Wearable thermoelectric materials and devices for self-powered electronic systems. *Adv. Mater.* **33**, 2102990 (2021).

12. A. Nozariasbmarz, H. Collins, K. Dsouza, M. H. Polash, M. Hosseini, M. Hyland, J. Liu, A. Malhotra, F. M. Ortiz, F. Mohaddes, V. P. Ramesh, Y. Sargolzaeiaval, N. Snouwaert, M. C. Özturk, D. Vashaee, Review of wearable thermoelectric energy harvesting: From body temperature to electronic systems. *Appl. Energy* **258**, 114069 (2020).

13. L. Miao, S. Zhu, C. Liu, J. Gao, Z. Zhang, Y. Peng, J.-L. Chen, Y. Gao, J. Liang, T. Mori, Comfortable wearable thermoelectric generator with high output power. *Nat. Commun.* **15**, 8516 (2024).

14. M. Zadan, A. Wertz, D. Shah, D. K. Patel, W. Zu, Y. Han, J. Gelorme, H. J. Mea, L. Yao, M. H. Malakooti, S. H. Ko, N. Kazem, C. Majidi, Stretchable thermoelectric generators for self-powered wearable health monitoring. *Adv. Funct. Mater.* **34**, 2404861 (2024).

15. H. M. Elmoughni, A. K. Menon, R. M. W. Wolfe, S. K. Yee, A textile-integrated polymer thermoelectric generator for body heat harvesting. *Adv. Mater. Technol.* **4**, 1800708 (2019).

16. Y. Lee, J. Park, J. Son, H. Y. Woo, J. Kwak, Degenerately doped semi-crystalline polymers for high performance thermoelectrics. *Adv. Funct. Mater.* **31**, 2006900 (2021).

17. C. K. Mytafides, L. Tzounis, G. Karalis, P. Formanek, A. S. Paipetis, High-power all-carbon fully printed and wearable SWCNT-based organic thermoelectric generator. *ACS Appl. Mater. Interfaces* **13**, 11151–11165 (2021).

18. B. Russ, A. Glaudell, J. J. Urban, M. L. Chabinyc, R. A. Segalman, Organic thermoelectric materials for energy harvesting and temperature control. *Nat. Rev. Mater.* **1**, 16050 (2016).

19. N. J. Pataki, N. Zahabi, Q. Li, P. Rossi, M. Cassinelli, M. Butti, M. Massetti, S. Fabiano, I. Zozoulenko, M. Caironi, A rolled organic thermoelectric generator with high thermocouple density. *Adv. Funct. Mater.* **34**, 2400982 (2024).

20. W. Zhou, Q. Fan, Q. Zhang, L. Cai, K. Li, X. Gu, F. Yang, N. Zhang, Y. Wang, H. Liu, W. Zhou, S. Xie, High-performance and compact-designed flexible thermoelectric modules enabled by a reticulate carbon nanotube architecture. *Nat. Commun.* **8**, 14886 (2017).

21. J. H. Song, J. Park, S. H. Kim, J. Kwak, Vitamin C-induced enhanced performance of PEDOT:PSS thin films for eco-friendly transient thermoelectrics. *ACS Appl. Mater. Interfaces* **15**, 2852–2860 (2023).

22. J. Jeong, S. Park, J. Park, J. Song, J. Kwak, Machine-learning-assisted process optimization for high-performance organic thermoelectrics. *Adv. Energy Mater.* **15**, 2403431 (2025).

23. S. Hwang, I. Jeong, J. Park, J.-K. Kim, H. Kim, T. Lee, J. Kwak, S. Chung, Enhanced output performance of all-solution-processed organic thermoelectrics: Spray printing and interface engineering. *ACS Appl. Mater. Interfaces* **12**, 26250–26257 (2020).

24. S. Kee, M. A. Haque, D. Corzo, H. N. Alshareef, D. Baran, Self-healing and stretchable 3D-printed organic thermoelectrics. *Adv. Funct. Mater.* **29**, 1905426 (2019).

25. N. Wen, Z. Fan, S. Yang, Y. Zhao, T. Cong, S. Xu, H. Zhang, J. Wang, H. Huang, C. Li, L. Pan, Highly conductive, ultra-flexible and continuously processable PEDOT:PSS fibers with high thermoelectric properties for wearable energy harvesting. *Nano Energy* **78**, 105361 (2020).

26. J. Park, J. G. Jang, K. Kang, S. H. Kim, J. Kwak, High thermoelectric performance in solution-processed semicrystalline PEDOT:PSS films by strong acid–base treatment: Limitations and potential. *Adv. Sci.* **11**, 2308368 (2024).

27. J. Park, Y. Lee, M. Kim, Y. Kim, A. Tripathi, Y.-W. Kwon, J. Kwak, H. Y. Woo, Closely packed polypyrroles via ionic cross-linking: Correlation of molecular structure–morphology–thermoelectric properties. *ACS Appl. Mater. Interfaces* **12**, 1110–1119 (2020).

28. Z. Liao, X. Zhou, G. Wei, S. Wang, C. Gao, L. Wang, Intrinsically self-healable and wearable all-organic thermoelectric composite with high electrical conductivity for heat harvesting. *ACS Appl. Mater. Interfaces* **14**, 43421–43430 (2022).

29. Y. Wang, Z. Zhou, J. Zhou, L. Shao, Y. Wang, Y. Deng, High-performance stretchable organic thermoelectric generator via rational thermal interface design for wearable electronics. *Adv. Energy Mater.* **12**, 2102835 (2022).

30. D. Kim, D. Ju, K. Cho, Heat-sink-free flexible organic thermoelectric generator vertically operating with chevron structure. *Adv. Mater. Technol.* **3**, 1700335 (2018).

31. N. Kim, S. Lienemann, I. Petsagkourakis, D. Alemu Mengistie, S. Kee, T. Ederth, V. Gueskine, P. Leclère, R. Lazzaroni, X. Crispin, K. Tybrandt, Elastic conducting polymer composites in thermoelectric modules. *Nat. Commun.* **11**, 1424 (2020).

32. T. Sun, J. L. Peavey, M. David Shelby, S. Ferguson, B. T. O'Connor, Heat shrink formation of a corrugated thin film thermoelectric generator. *Energy Convers. Manag.* **103**, 674–680 (2015).

33. K. T. Park, Y. S. Cho, I. Jeong, D. Jang, H. Cho, Y. Choi, T. Lee, Y. Ko, J. Choi, S. Y. Hong, M.-W. Oh, S. Chung, C. R. Park, H. Kim, Highly integrated, wearable carbon-nanotube-yarn-based thermoelectric generators achieved by selective inkjet-printed chemical doping. *Adv. Energy Mater.* **12**, 2200256 (2022).

34. W. Ren, Y. Sun, D. Zhao, A. Aili, S. Zhang, C. Shi, J. Zhang, H. Geng, J. Zhang, L. Zhang, J. Xiao, R. Yang, High-performance wearable thermoelectric generator with self-healing, recycling, and Lego-like reconfiguring capabilities. *Sci. Adv.* **7**, eabe0586 (2021).

35. K. Nan, S. D. Kang, K. Li, K. J. Yu, F. Zhu, J. Wang, A. C. Dunn, C. Zhou, Z. Xie, M. T. Agne, H. Wang, H. Luan, Y. Zhang, Y. Huang, G. J. Snyder, J. A. Rogers, Compliant and stretchable

thermoelectric coils for energy harvesting in miniature flexible devices. *Sci. Adv.* **4**, eaau5849 (2018).

36. Z. Guo, Y. Yu, W. Zhu, Q. Zhang, Y. Liu, J. Zhou, Y. Wang, J. Xing, Y. Deng, Kirigami-based stretchable, deformable, ultralight thin-film thermoelectric generator for bodyNET application. *Adv. Energy Mater.* **12**, 2102993 (2022).

37. A. G. Rösch, A. Gall, S. Aslan, M. Hecht, L. Franke, M. M. Mallick, L. Penth, D. Bahro, D. Friderich, U. Lemmer, Fully printed origami thermoelectric generators for energy-harvesting. *NPJ Flex. Electron.* **5**, 1 (2021).

38. K. Uchida, J. P. Heremans, Thermoelectrics: From longitudinal to transverse. *Joule* **6**, 2240–2245 (2022).

39. W. Zhou, K. Yamamoto, A. Miura, R. Iguchi, Y. Miura, K.-i. Uchida, Y. Sakuraba, Seebeck-driven transverse thermoelectric generation. *Nat. Mater.* **20**, 463–467 (2021).

40. J. Xu, W. A. Phelan, C.-L. Chien, Large anomalous nernst effect in a van der waals ferromagnet Fe_3GeTe_2. *Nano Lett.* **19**, 8250–8254 (2019).

41. T. Chen, S. Minami, A. Sakai, Y. Wang, Z. Feng, T. Nomoto, M. Hirayama, R. Ishii, T. Koretsune, R. Arita, S. Nakatsuji, Large anomalous Nernst effect and nodal plane in an iron-based kagome ferromagnet. *Sci. Adv.* **8**, eabk1480 (2022).

42. A. Sakai, Y. P. Mizuta, A. A. Nugroho, R. Sihombing, T. Koretsune, M.-T. Suzuki, N. Takemori, R. Ishii, D. Nishio-Hamane, R. Arita, P. Goswami, S. Nakatsuji, Giant anomalous Nernst effect and quantum-critical scaling in a ferromagnetic semimetal. *Nat. Phys.* **14**, 1119–1124 (2018).

43. K. Uchida, M. Ishida, T. Kikkawa, A. Kirihara, T. Murakami, E. Saitoh, Longitudinal spin Seebeck effect: From fundamentals to applications. *J. Phys. Condens. Matter* **26**, 343202 (2014).

44. K. Uchida, S. Takahashi, K. Harii, J. Ieda, W. Koshibae, K. Ando, S. Maekawa, E. Saitoh, Observation of the spin Seebeck effect. *Nature* **455**, 778–781 (2008).

45. A. Sakai, S. Minami, T. Koretsune, T. Chen, T. Higo, Y. Wang, T. Nomoto, M. Hirayama, S. Miwa, D. Nishio-Hamane, F. Ishii, R. Arita, S. Nakatsuji, Iron-based binary ferromagnets for transverse thermoelectric conversion. *Nature* **581**, 53–57 (2020).

46. K. Uchida, H. Adachi, T. Kikkawa, A. Kirihara, M. Ishida, S. Yorozu, S. Maekawa, E. Saitoh, Thermoelectric generation based on spin seebeck effects. *Proc. IEEE* **104**, 1946–1973 (2016).

47. Y. Ma, X. Ren, Y. Zou, W. Zhao, D. Wang, Z. Ji, J. Fan, C. Yan, L. Xiang, G. Ge, X. Dai, F. Zhang, T. Lei, H. Sirringhaus, C.-a. Di, D. Zhu, Observation of anomalously large Nernst effects in conducting polymers. *Nat. Commun.* **16**, 1435 (2025).

48. S. Roychowdhury, A. M. Ochs, S. N. Guin, K. Samanta, J. Noky, C. Shekhar, M. G. Vergniory, J. E. Goldberger, C. Felser, Large room temperature anomalous transverse thermoelectric effect in kagome antiferromagnet YMn_6Sn_6. *Adv. Mater.* **34**, 2201350 (2022).

49. T. Kikkawa, E. Saitoh, Spin seebeck effect: Sensitive probe for elementary excitation, spin correlation, transport, magnetic order, and domains in solids. *Annu. Rev. Condens. Matter Phys.* **14**, 129–151 (2023).

50. J. Wei, M. Liao, A. Ma, Y. Chen, Z. Duan, X. Hou, M. Li, N. Jiang, J. Yu, Enhanced thermal conductivity of polydimethylsiloxane composites with carbon fiber. *Compos. Commun.* **17**, 141–146 (2020).

51. S. Wang, G. Zuo, J. Kim, H. Sirringhaus, Progress of conjugated polymers as emerging thermoelectric materials. *Prog. Polym. Sci.* **129**, 101548 (2022).

52. S. Wang, W. Zhu, I. E. Jacobs, W. A. Wood, Z. Wang, S. Manikandan, J. W. Andreasen, H.-I. Un, S. Ursel, S. Peralta, S. Guan, J.-C. Grivel, S. Longuemart, H. Sirringhaus, Enhancing the thermoelectric properties of conjugated polymers by suppressing dopant-induced disorder. *Adv. Mater.* **36**, 2314062 (2024).

53. M. G. Cooper, B. B. Mikic, M. M. Yovanovich, Thermal contact conductance. *Int. J. Heat Mass Transf.* **12**, 279–300 (1969).

54. C. Creton, M. Ciccotti, Fracture and adhesion of soft materials: A review. *Rep. Prog. Phys.* **79**, 046601 (2016).

55. S. H. Kim, S. Jung, I. S. Yoon, C. Lee, Y. Oh, J.-M. Hong, Ultrastretchable conductor fabricated on skin-like hydrogel–elastomer hybrid substrates for skin electronics. *Adv. Mater.* **30**, 1800109 (2018).

56. J. H. Song, J. Jeong, J. Park, G. Park, I. Imae, J. Kwak, Enhanced performance and stability of PEDOT:PSS thin-film thermoelectrics by de-doping with nitrogen-doped graphene quantum dots. *ACS Appl. Electron. Mater.* **6**, 6313–6321 (2024).

57. E. Pop, V. Varshney, A. K. Roy, Thermal properties of graphene: Fundamentals and applications. *MRS Bulletin* **37**, 1273–1281 (2012).

58. L. T. Su, J. E. Chung, D. A. Antoniadis, K. E. Goodson, M. I. Flik, Measurement and modeling of self-heating in SOI nMOSFET's. *IEEE Trans. Electron Devices* **41**, 69–75 (1994).

59. J.-Y. Sun, X. Zhao, W. R. K. Illeperuma, O. Chaudhuri, K. H. Oh, D. J. Mooney, J. J. Vlassak, Z. Suo, Highly stretchable and tough hydrogels. *Nature* **489**, 133–136 (2012).

60. R. S. Rivlin, A. G. Thomas, Rupture of rubber. I. Characteristic energy for tearing. *J. Polym. Sci.* **10**, 291–318 (1953).

61. G. Kafkopoulos, C. J. Padberg, J. Duvigneau, G. J. Vancso, Adhesion engineering in polymer–metal comolded joints with biomimetic polydopamine. *ACS Appl. Mater. Interfaces* **13**, 19244–19253 (2021).

62. M. H. Ho, Q. van Hilst, X. Cui, Y. Ramaswamy, T. Woodfield, J. Rnjak-Kovacina, S. G. Wise, K. S. Lim, From adhesion to detachment: Strategies to design tissue-adhesive hydrogels. *Adv. NanoBiomed Res.* **4**, 2300090 (2024).

63. H. Massé, É. Arquis, D. Delaunay, S. Quilliet, P. H. Le Bot, Heat transfer with mechanically driven thermal contact resistance at the polymer–mold interface in injection molding of polymers. *Int. J. Heat Mass Transf.* **47**, 2015–2027 (2004).

64. M. C. Rajagopal, T. Man, A. Agrawal, G. Kuntumalla, S. Sinha, Intrinsic thermal interfacial resistance measurement in bonded metal–polymer foils. *Rev. Sci. Instrum.* **91**, 104901 (2020).

65. B. Wei, W. Luo, J. Du, Y. Ding, Y. Guo, G. Zhu, Y. Zhu, B. Li, Thermal interface materials: From fundamental research to applications. *SusMat* **4**, e239 (2024).

66. M. Ikhlas, T. Tomita, T. Koretsune, M.-T. Suzuki, D. Nishio-Hamane, R. Arita, Y. Otani, S. Nakatsuji, Large anomalous Nernst effect at room temperature in a chiral antiferromagnet. *Nat. Phys.* **13**, 1085–1090 (2017).

67. S. N. Guin, K. Manna, J. Noky, S. J. Watzman, C. Fu, N. Kumar, W. Schnelle, C. Shekhar, Y. Sun, J. Gooth, C. Felser, Anomalous Nernst effect beyond the magnetization scaling relation in the ferromagnetic Heusler compound Co_2MnGa. *NPG Asia Mater.* **11**, 16 (2019).

68. M. Li, H. Pi, Y. Zhao, T. Lin, Q. Zhang, X. Hu, C. Xiong, Z. Qiu, L. Wang, Y. Zhang, J. Cai, W. Liu, J. Sun, F. Hu, L. Gu, H. Weng, Q. Wu, S. Wang, Y. Chen, B. Shen, Large anomalous nernst effects at room temperature in Fe3Pt thin films. *Adv. Mater.* **35**, 2301339 (2023).

69. G. Lopez-Polin, H. Aramberri, J. Marques-Marchan, B. I. Weintrub, K. I. Bolotin, J. I. Cerdá, A. Asenjo, High-power-density energy-harvesting devices based on the anomalous nernst effect of Co/Pt magnetic multilayers. *ACS Appl. Energy Mater.* **5**, 11835–11843 (2022).

70. W. Zhou, Y. Sakuraba, Heat flux sensing by anomalous Nernst effect in Fe–Al thin films on a flexible substrate. *Appl. Phys. Express* **13**, 043001 (2020).
